# Protein phosphatase 1 regulatory subunit 15 A (PPP1R15A) promoted the progression of gastric cancer by activating cell autophagy under energy stress

**DOI:** 10.1186/s13046-025-03320-y

**Published:** 2025-02-13

**Authors:** Yingnan Cui, Xueyuan Cao, Yangyu Zhang, Chenhao Fu, Dongming Li, Yuanlin Sun, Yuzheng Zhang, Tingshuang Xu, Tetsuya Tsukamoto, Donghui Cao, Jing Jiang

**Affiliations:** 1 Division of Clinical Epidemiology, The First Hospital of Jilin University, Changchun, Jilin China; 2 Department of Gastric and Colorectal Surgery, General Surgery Center, The First Hospital of Jilin University, Changchun, Jilin China; 3 Doctor of excellence program (DEP), The First Hospital of Jilin University, Changchun, Jilin China; 4 Department of Hospital Infection Management, China-Japan Union Hospital of Jilin University, Changchun, Jilin China; 5 Core facility of The First Hospital of Jilin University, Changchun, Jilin China; 6 Department of Diagnostic Pathology I, School of Medicine, Fujita Health University, Toyoake, Japan

**Keywords:** Gastric cancer, Energy stress, Autophagy, Protein Phosphatase 1 Regulatory Subunit 15A (PPP1R15A), JUN

## Abstract

**Background:**

Glucose metabolism plays a critical role in tumor progression. When glucose intake is insufficient and the tumor’s growth rate exceeds its energy supply, tumor cells typically adapt and overcome the energy stress through compensatory mechanisms to maintain the survival of tumor cells, which may also be related to tumor recurrence or metastasis.

**Methods:**

Different concentrations of glucose were selected as the basis for the energy stress model of gastric cancer. Then CCK-8 and flow cytometry were used to detect its effects on cell proliferation, apoptosis, and cell cycle. Differentially expressed genes (DEGs) were screened by RNA sequencing and the regulated pathways were identified by gene set enrichment analysis. The regulatory relationship between the gene PPP1R15A and its transcription factor JUN was proved by ChIP-qPCR and dual-luciferase reporter assay. The gain and loss of function assays were conducted to examine the effects of PPP1R15A under energy stress in vivo and in vitro. Potential regulatory mechanisms of PPP1R15A were further analyzed through a combination of online databases, RNA sequencing, and metabolite sequencing. The regulation of PPP1R15A on cell autophagy under energy stress was detected by western blot, transmission electron microscope, mRFP-GFP-LC3 adenovirus and laser scanning confocal microscopy.

**Results:**

PPP1R15A and the transcription factor JUN were significantly upregulated by glucose deprivation (0 mM vs. 25 mM), JUN combined with the promoter of PPP1R15A and activated its expression. Both PPP1R15A and JUN were highly expressed in gastric cancer tissues and were independent risk factors for prognosis in the gastric cancer cohort. Overexpression of PPP1R15A promoted cell proliferation, inhibited apoptosis, and was involved in cell cycle arrest. Further RNA and metabolite sequencing suggested that PPP1R15A was associated with cell autophagy. In vitro experiments confirmed that both glucose deprivation and overexpression of PPP1R15A promoted the biosynthesis of autolysosome and autophagosome, and activated the cleavage of LC3 complex in gastric cancer cells. Moreover, PPP1R15A knockdown inhibited cell autophagy induced by glucose deprivation.

**Conclusions:**

PPP1R15A sustained the survival of gastric cancer cells by regulating autophagy under energy stress to resist or adapt to harsh environments.

**Supplementary Information:**

The online version contains supplementary material available at 10.1186/s13046-025-03320-y.

## Background

Gastric cancer (GC) is prevalent globally, with the fifth-highest incidence and mortality rate of all malignant tumors worldwide in 2022 and 359,000 new cases in China, ranking fifth among all malignant tumors. Additionally, the death cases were 260,000, ranking third among all malignant tumors [1]. Recurrence and metastasis are the leading cause of poor GC prognosis [2, 3]. Clarifying the mechanisms of recurrence and metastasis will provide patients with more chances to survive.

The occurrence and progression of solid tumors are inseparable from glucose metabolism. Metabolic stress occurs when tumor growth exceeds its glucose supply (glucose deprivation) [4]. Vincent revealed that the glucose concentration in normal tissues is approximately 3–10 times higher than that in tumor tissues [5], indicating the presence of energy stress in tumor tissues. Several studies demonstrated that the glycolysis-related genes were crucial to tumor cell survival under energy stress [6], despite numerous metabolic enzymes investigated as potential targets for cancer treatment [7], and the presence of adverse effects limits the efficacy of metabolic therapy [8].

Autophagy plays different roles in various tumor development stages. Autophagy, as a quality control system, inhibits tumor formation and impedes cancer progression in the early stages of the tumor. Autophagy may transform into a dynamic degradation and recycling system, maintaining the growth, and improving the aggressiveness of cancer cells [9]. Several studies revealed autophagy endowed cells with the ability to survive under nutrient restriction conditions [10–12]. However, the role of autophagy in adapting to energy stress warrants clarification.

The present study developed an energy stress model to observe the effects of glucose deprivation on cell viability, apoptosis, and cell cycle. Multiple sequencing methods and bioinformatic analysis revealed that cell autophagy was involved in the adaptation mechanism of GC cells to energy stress. Additionally, the regulatory subunit 15 A of protein phosphatase 1 (PPP1R15A), which is induced by multiple stressors, and its potential transcription factor c-Jun were crucial for cell autophagy and cell survival under the harsh environment.

## Methods

### Cell culture and reagents

The human GC cell lines, including AGS, BGC-823, HGC-27, and SGC-7901, were cultured in Roswell Park Memorial Institute (RPMI)-1640 complete medium (supplemented with 10% fetal bovine serum and 1% penicillin/streptomycin solution) (Biological Industries). The immortalized gastric epithelial cell line GES-1 was utilized as a control. Cells were washed twice with phosphate-buffered saline (PBS) and then incubated in a glucose-free complete RPMI-1640 medium (Gibco) supplemented with different concentrations of glucose (1, 11, 25 mM) (Solarbio) to induce energy stress. The cells were incubated with 5% CO_2_ at 37 °C.

### Patients and tissue specimens

All the patients were histologically diagnosed with GC in the Department of Gastric and Colorectal Surgery of the First Hospital of Jilin University (Changchun, China). Fresh GC (Cancer) and the paired adjacent control (Normal) tissues were categorized into two: part one involves freezing nucleic acid and protein extraction and fixing the other with neutral formalin for 12 h and part two involves preparing paraffin-embedded tissue chips and slicing them as 4 μm by professional technicians in the department of pathology. The characteristics of GC patients are shown in Supplementary Table 1.

### Xenografted tumor model

HGC-27 cells (1 × 10^7^) stably expressing PPP1R15A (OE, overexpression; EV, empty vector) were suspended in 250 µl of PBS with matrigel (3:1), and then injected into BALB/C-nu mice (6–7 weeks old, female) subcutaneously. A digital caliper was used to measure tumor volumes every three days. Mice were sacrificed and tumor tissues were collected at the termination of the experiment. Tumor volume (mm^3^) was calculated by the formula: tumor volume = (length × width^2^)/2, where length denotes the longest tumor diameter and width represents the perpendicular tumor diameter.

### Omics sequencing

All sequencing involved in this study was performed in LC-Bio Technology co.ltd., (Hangzhou, China), including RNA-seq (three replicates in each group) for the energy stress model, metabolite-seq (six replicates in each group), and RNA-seq (three replicates in each group) for PPP1R15A overexpressed HGC-27 cells. The Differentially expressed genes (DEGs) in RNA-seq for the energy stress model were detected (log2fc > 1 or < − 1, *P* < 0.05). The DEGs in RNA-seq and metabolite-seq for PPP1R15A overexpressed cells were detected (*P* < 0.05).

### Plasmid and siRNA transfection

GenePharma (Shanghai, China) provided the siRNA sequences and overexpression vector for PPP1R15A, and Zebrafish Biotech (Nanjing, China) supplied the overexpression vector for JUN. The transfection reagent Lipofectamine 3000 (Invitrogen) was used following the manufacturer’s instructions. Cells were transfected and gathered for further experiments, and the efficacy of interference or overexpression was evaluated by quantitative real-time polymerase chain reaction (RT-qPCR) and Western blot. Supplementary Table 2 shows the detailed siRNA sequences.

### shRNA and overexpression lentiviral infection

Cells were infected with lentiviral particles containing PPP1R15A-targeted shRNA (GenePharma) or overexpression vector (Zebrafish Biotech) for stable PPP1R15A knockdown or overexpression, followed by stable expression cells selection using 2 µg/mL of puromycin for 14 days. Supplementary Table 3 presents the detailed shRNA sequences.

### Cell viability

Cells were plated in 96-well plates at a density of 5,000 cells per well, and cultured for 24 h, and the cell viability was tested with the Cell Counting Kit-8 (CCK-8, US EVERBRIGHT, Suzhou, China) following the manufacturer’s instructions.

### Colony formation assay

Cells were plated in a 6-well plate at a density of 200 cells per well, and cultured for approximately 14–21 days. Cells were rinsed twice with PBS, fixed with methanol for 20 min, and then stained with Giemsa for 20 min after removing the medium. Clones were considered effective if they contained > 50 cells. ImageJ was used to process and quantify images. This experiment was conducted three times for validation.

### Flow cytometry

The apoptosis and cell cycle distribution were detected with the YF^®^488-Annexin V/PI Apoptosis Analysis Kit and the Cell Cycle Analysis Kit (US EVERBRIGHT, Suzhou, China) independently following the manufacturer’s instructions. The cells were evaluated by flow cytometry (BD Biosciences, USA) with three replicates, and FlowJo was used for data analysis.

### Quantitative real-time PCR

FastPure Cell/Tissue Total RNA Isolation Kit (Vazyme, Nanjing, China) was used for total RNA extraction. Subsequently, MonScript™ RTIII All-in-One Mix (Monad, Suzhou, China) was utilized for cDNA synthesis. Quantitative real-time PCR analysis was carried out utilizing MonAmp™ ChemoHS qPCR Mix (Monad). Relative RNA levels were identified by the standard 2^−ΔΔCt^ method. Supplementary Table 4 shows the primers.

### Chromatin immunoprecipitation (ChIP)-qPCR

The ChIP experiment was conducted using a ChIP kit (CST, USA) following the manufacturer’s instructions. RT-qPCR was then used to evaluate the ChIP signal, and presented the data using the %Input method. Supplementary Table 5 presents the specific primers used in the ChIP-qPCR analysis.

### Dual-luciferase reporter assay

Cells were seeded in 96-well plates one day before transfection and then cotransfected with PPP1R15A promoter plasmid (Promoter) and JUN overexpression plasmid (JUN) using Lipofectamine 3,000 reagent, and the control plasmid without promoter (NC) and the empty vector (Vector) were utilized as control. Luciferase activity analysis was performed 48 h after using a Dual-Luciferase Reporter Gene Assay Kit (Genbiotech, Beijing, China) detected by Fluorescence/Multi-Detection Microplate Reader (BioTek, USA). The firefly luciferase activities were normalized to Renilla luminescence in each cell well.

### Western blot (WB)

Proteins were extracted from the cells utilizing a strong RIPA lysis buffer supplemented with a protease inhibitor cocktail (Coolaber, Beijing, China), separated by SDS-PAGE (Epizyme, Shanghai, China), and then transferred to the PVDF membrane (Millipore). The PVDF membranes were incubated with specific antibodies at 4 °C overnight after incubation with the secondary antibody (Absin, Shanghai, China) at room temperature for 1 h. Protein signals were detected by Omni-ECL™ Pico Light Chemiluminescence Kit (Epizyme, Shanghai, China). Supplementary Table 6 presents detailed information about antibodies.

### Immunohistochemistry (IHC)

This study included 246 pairs of tumors and adjacent tissues from patients with GC. Antigen repair was performed in sodium citrate buffer (pH: 6.0) after deparaffinization and rehydration. Endogenous peroxidase activity was quenched before proceeding with primary and secondary antibody incubation. DAB method was utilized for color development and then the slices were counterstained with hematoxylin. Protein staining intensity was categorized into 0, 1, 2, and 3. The histochemistry-score (H-Score) method was used for quantitative analysis, combined with the staining percentage Pi of each intensity. The calculation method was H-Score = Σ (i × Pi). This study used the median grouping method. The relevant reagents were purchased from Fuzhou MaiXin Biotechnology. Supplementary Table 6 presents detailed information about antibodies.

### mRFP-GFP-LC3B adenovirus infection

Cells were infected with mRFP-GFP-LC3 adenovirus (Hanheng, Shanghai, China) and incubated for 48 h to assess tandem fluorescent LC3 puncta. Subsequently, cells were washed with 1 × PBS and treated as required. The cells were then observed under a laser scanning confocal microscope. The mRFP in the virus labels and tracks LC3. The weakening of GFP indicates the lysosome and autophagosome fusion to form autolysosome. That is because the GFP fluorescent protein is sensitive to acidity, and only red fluorescence can be detected when the GFP fluorescence is quenched after the autophagosome and lysosome fusion. The red and green fluorescence merge after microscope imaging, and the yellow spots that appear after the merge are just autophagosomes. Red spots indicate autolysosomes, and the intensity of autophagic flow can be observed by counting different colored spots.

### Transmission electron microscopy

Cells in different groups were collected and fixed using 2.5% glutaraldehyde at room temperature and rinsed three times using 0.1 M phosphate-buffered PB (pH: 7.4) for 15 min each time. The cells were refixed using 1% osmium acid and 0.1 M phosphate-buffered PB for 2 h at room temperature and rinsed thrice using 0.1 M PB. The cells were then dehydrated using 50%, 70%, 80%, 90%, 95%, and 100% alcohol–100% acetone for 15 min each. Additionally, the cells were permeated, embedded using Epon812 embedding medium, sliced 60–80 nm ultra-thin section, double stained using uranium-lead double staining solution, and observed and analyzed under a transmission electron microscope. Jijia Biotech (Shenyang, China) provided the services.

### Bioinformatics

The data were downloaded from The Cancer Genome Atlas Program (TCGA) (https://portal.gdc.cancer.gov/), Gene Expression Omnibus (GEO) (https://www.ncbi.nlm.nih.gov/geo/), and Genotype-Tissue Expression (GTEx) (https://gtexportal.org/home/datasets). The TCGA database included clinical information from 375 patients with GC and 32 healthy controls. The characteristics of GC patients are shown in Supplementary Table 7. The GEO database included clinical information from 875 patients with GC and the GTEx database included 359 healthy controls. The GSE13548 dataset contains sequencing results of GC cell MKN-74 under glucose-free conditions.

The Gene Ontology (GO) and Kyoto Encyclopedia of genes and genomes (KEGG) pathway enrichment analyses were conducted with R package ggplot. Gene Set Enrichment Analysis (GSEA) was performed with GSEA version 4.1.0 and the results were presented by the Hiplot website (https://hiplot.com.cn/). ACLBI (https://www.aclbi.com/static/) was also used for online bioinformatics analyses. Data presentation and gene enrichment analysis are partly conducted on the website (https://www.xiantaozi.com/). The prediction of transcription factor was performed on the websites of JASPAR (https://jaspar.elixir.no/) and ALGGEN-PROMO (http://alggen.lsi.upc.es/cgi-bin/promo_v3/promo/promoinit.cgi?dirDB=TF_8.3/).

### Statistical analysis

The *t*-test was used for analyzing two-group comparisons, whereas comparisons involving more than two groups (> 2) were conducted using two-way analysis of variance. Results were presented as the mean ± standard deviation (SD) of multiple independent experiments. GraphPad Prism 9.0 was used for statistical analysis, with significance set at *P*-values of < 0.05. Each experiment was conducted at least thrice. Protein distribution in different groups was indicated by frequency and composition ratio (%), and the chi-squared test and Fisher’s exact test were used to compare differences in protein distribution across different groups. Kaplan–Meier survival curve, log-rank test, and Cox proportional-hazards model were used to analyze the association between protein expression levels and overall survival in patients with GC.

## Results

### Energy stress-induced cell autophagy in GC

Different glucose concentrations (0 mM, 1 mM, 11 mM, and 25 mM) were selected to develop the energy stress model, considering the results of the literature review. RT-qPCR revealed that gene expressions related to the glucose metabolic pathway, such as glucose-6-phosphate dehydrogenase (HK1), lactate dehydrogenase (LDHA), pyruvate dehydrogenase kinase 1 (PDK1), and glucose transporter member 1 (GLUT1), in GC cells were significantly increased under glucose starvation (1 mM) especially deprivation (0 mM), indicating that the energy stress model was successfully established (Supplementary Fig. 1A). Further CCK-8 assays revealed that AGS and HGC-27 cell proliferation were inhibited in glucose starvation and deprivation medium (SFig. 1B). Additionally, flow cytometry detected increased apoptosis (SFig. 1 C) and the arrested cell cycles in the G0/G1 phase (SFig. 1D) under glucose starvation and deprivation. Glucose-free medium (0 mM) was then used to develop the energy stress model in the following research, and 25 mM glucose was set as control.

**Fig. 1:**
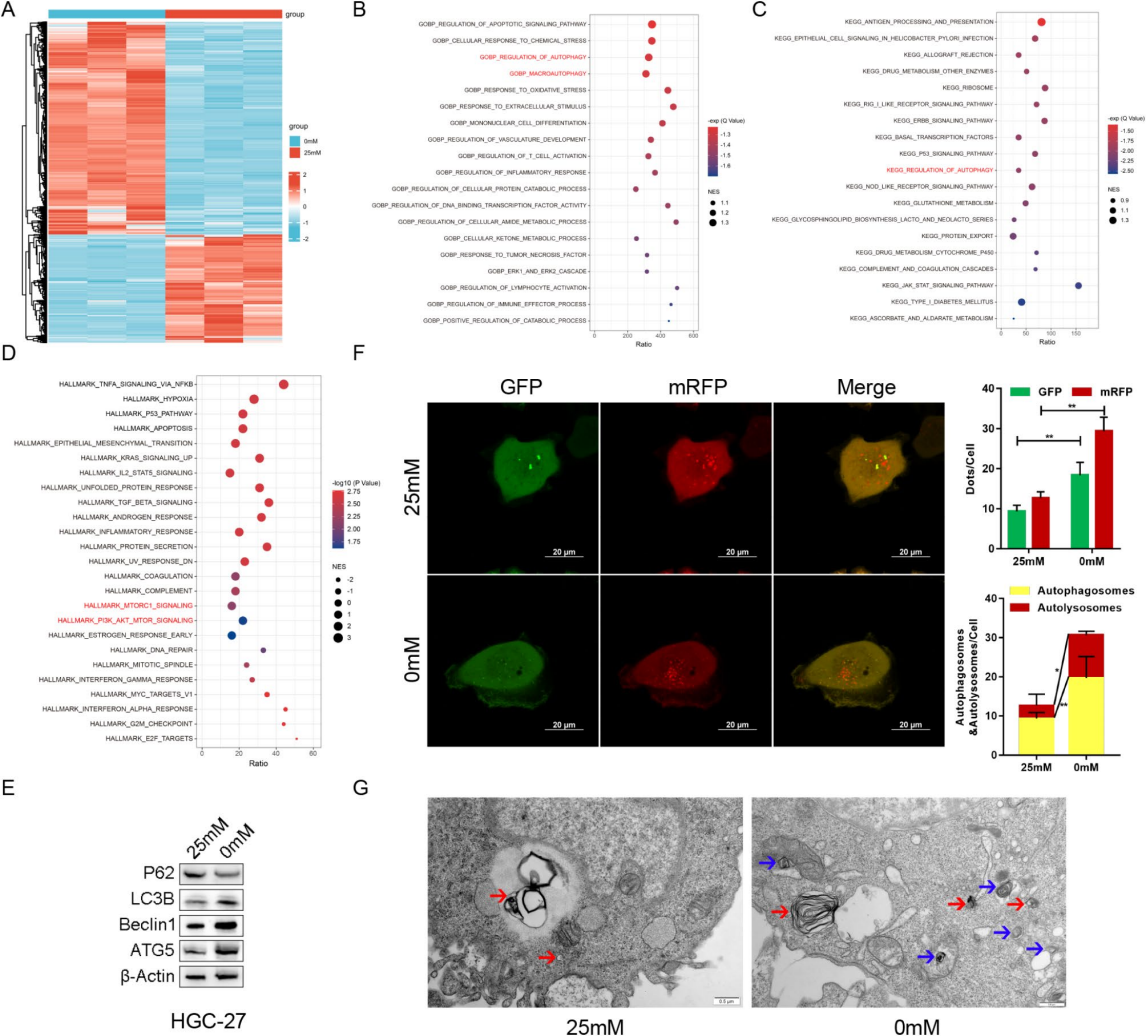
Energy stress-induced cell autophagy in gastric cancer (GC). **(A)** Differential expression genes (DEGs) were detected in HGC-27 under glucose deprivation for 24 h using RNA-seq. **(B)** Gene Ontology (GO) enrichment analysis of DEGs. **(C)** Kyoto Encyclopedia of genes (KEGG) pathway enrichment analysis of DEGs. **(D)** HALLMARK gene set enrichment analysis of DEGs. **(E)** Autophagy-related protein expressions detected in HGC-27. **(F)** Autophagosome and autolysosome formation detected in HGC-27 using mRFP-GFP-LC3B adenovirus infection by laser scanning confocal microscopy. The red spots indicated autolysosomes, whereas the yellow spots denoted autophagosomes. **(G)** Autophagosome and autolysosome formation in HGC-27 detected by transmission electron microscopy. The blue arrows represented autophagosomes and autophagy prophase, whereas the red arrows indicated autolysosomes

RNA-seq was conducted on HGC-27 cells cultured in the glucose-free medium (0 mM) to look for the key genes and possible mechanisms of energy stress. Contrasted with the control group (25 mM), 3081 DEGs were detected (log_2_fc > 1 or < − 1, *P* < 0.05), including 1,053 downregulated genes and 2,028 upregulated genes (Fig. 1A). GO, KEGG, and HALLMARK pathway enrichment analyses concurrently revealed that cell autophagy was regulated by energy stress (Fig. 1B-D). Additionally, WB identified upregulated autophagy marker expression, including MAP1LC3B, BECN1, and ATG5, and improved LC3 cleavage, but decreased SQSTM1 (p62) both in HGC-27 and AGS under energy stress (Fig. 1E, SFig. 2 A). Additionally, mRFP-GFP-LC3B autophagy double labeled adenovirus was utilized to infect HGC-27, and the intensity of red and yellow fluorescence significantly increased under energy stress, indicating autophagosome and autolysosome formation (Fig. 1F, SFig. 2B). Further electron microscopic examination revealed that energy stress increased autophagosome content in bilayer or multilayer membranes (blue arrows) and autolysosomes in monolayer membranes (red arrows) (Fig. 1G), indicating that energy stress stimulates the autophagy level.

### PPP1R15A and transcription factor JUN were involved in the adaption of GC to energy stress

**Fig. 2:**
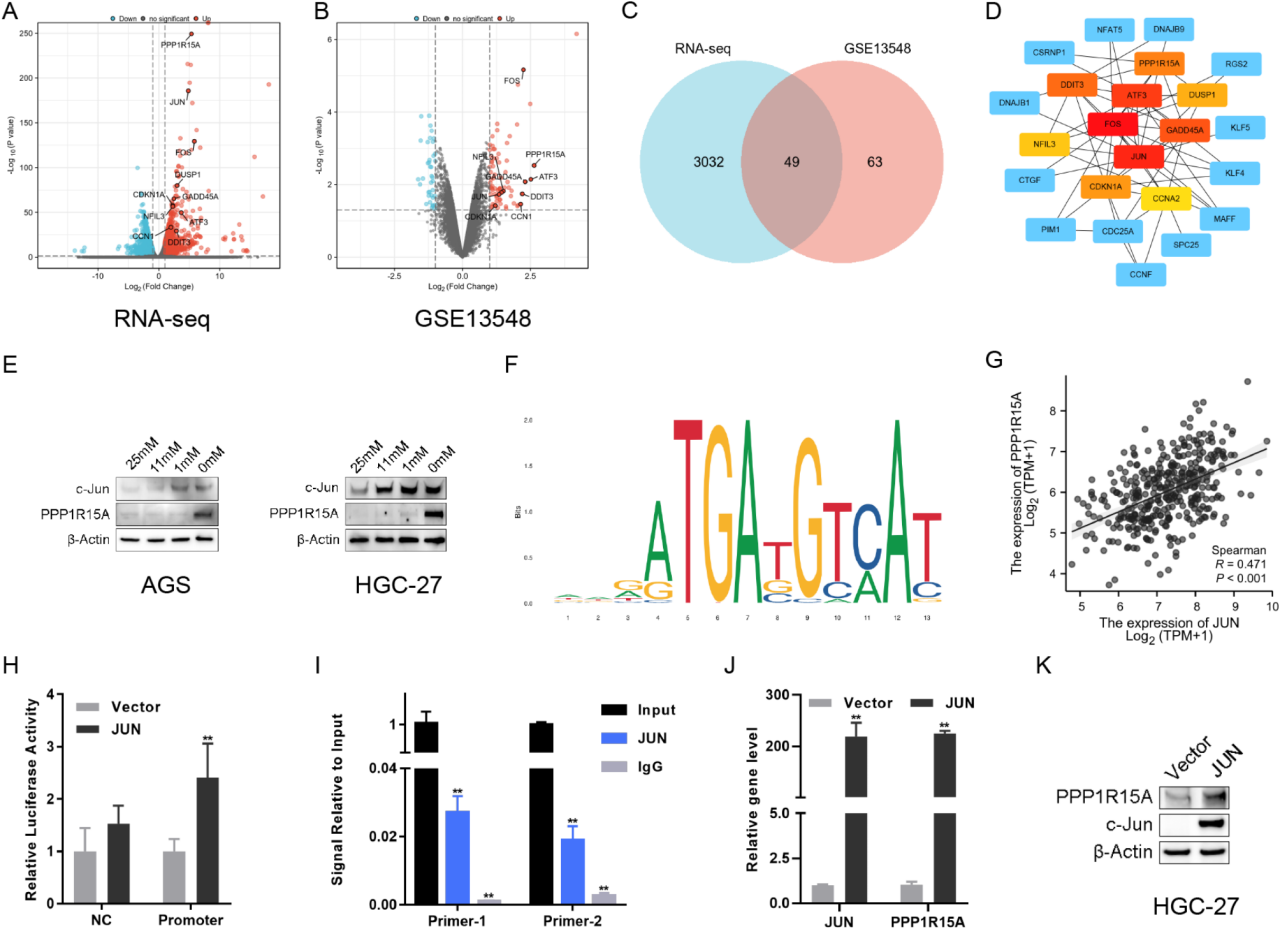
PPP1R15A and transcription factor JUN involved in the adaption of GC to energy stress. **(A)** Volcanic map of RNA-seq data of HGC-27 under glucose deprivation. The red dots indicated upregulated genes, whereas the blue dots denoted downregulated genes. **(B)** Volcanic map of GSE13548 sequencing data. The red dots indicated upregulated genes, whereas the blue dots represented downregulated genes. **(C)** The intersection of DEGs between RNA-seq and GSE13548. **(D)** Hub genes involved in energy stress using cytoHubba, a plugin in Cytoscape software. **(E)** The protein expression levels of c-Jun and PPP1R15A in AGS and HGC-27 cells under different glucose concentrations for 24 h. **(F)** The binding site of transcription factor c-Jun and PPP1R15A promoter was predicted on the JASPAR website. **(G)** The correlation between PPP1R15A and JUN expressions in TCGA-STAD. **(H)** Dual-luciferase reporter gene assay after the transfection of the JUN overexpression vector (JUN) and empty vector (Vector) used as control. **(I)** ChIP-qPCR using a c-Jun antibody and IgG. (**J**, **K**) The mRNA **(J)** and protein **(K)** expression levels of PPP1R15A and JUN after the transfection of the JUN overexpression vector and empty vector used as control

Besides the above RNA-seq data (Fig. 2A), GSE13548, including the RNA-seq data of MKN-74 cell under glucose deprivation, was analyzed (Fig. 2B). The HALLMARK gene set was utilized for GSEA, and the results revealed that DEGs in two sequencing data similarly involved in several pathways, including hypoxia, TNFα-NFκB, P53, apoptosis, and unfolded protein response (SFig. 3 A-B), confirming the general applicability of the energy stress model. Additionally, 49 common DEGs were observed in two sequencing data (Fig. 2C). These genes were imported into the STRING website to map the protein interaction network and then into the Cytoscape software to identify relevant key genes following the maximal clique centrality. The results revealed that the hub genes included transcription factors JUN, FOS, ATF3, and GADD family genes, including GADD45A, PPP1R15A (GADD34), DDIT3 (GADD153), etc. (Fig. 2D). An inducible gene PPP1R15A sensing bioenergetic stress in multiple cells, was selected to study its role under energy stress [13]. Indeed, WB indicated the upregulated PPP1R15A expression in multiple GC cells under glucose starvation. Concurrently, transcription factor c-Jun was activated under energy stress (Fig. 2E).

Transcription factor c-Jun binds with the PPP1R15A promoter with the potential binding sequences shown in Fig. 2F according to the prediction results of the JASPAR, and the expression levels of the two genes were positively correlated in the TCGA-stomach adenocarcinoma (STAD) database (*r* = 0.471, *P* < 0.001) (Fig. 2G). Additionally, the dual-luciferase reporter assay was used to verify the regulation effects of c-Jun on PPP1R15A. The results revealed that c-Jun overexpression improved the fluorescence value and promoter activity of the PPP1R15A both in HGC-27 and AGS cells (Fig. 2H, SFig. 4 A), indicating c-Jun tans-activated PPP1R15A directly. Furthermore, primers were designed for the predicted binding region (CGTGACGTCAGC), and ChIP-PCR confirmed that c-Jun binds with PPP1R15A promoter (Fig. 2I). The mRNA and protein expression levels of PPP1R15A were increased after JUN overexpression in HGC-27 and AGS (Fig. 2J–K, SFig. 4B–C). The above results indicated that c-Jun combined with PPP1R15A the promoter and upregulated its expression directly.

### The prognostic value of PPP1R15A and JUN in GC

**Fig. 3:**
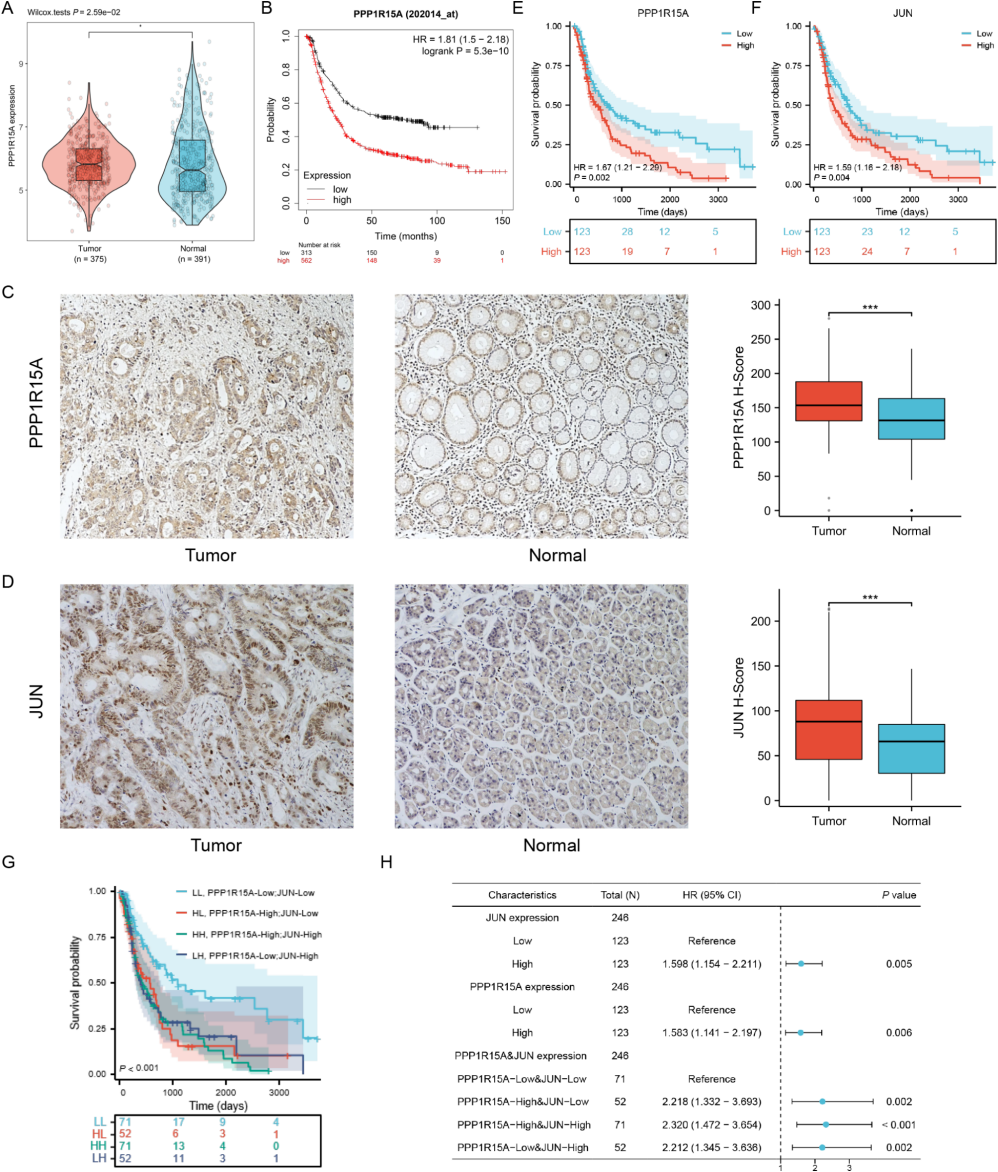
The prognostic value of PPP1R15A and JUN in GC. **(A)** The mRNA expression of PPP1R15A in TCGA-STAD and GTEx. **(B)** The prognostic value of PPP1R15A mRNA levels in the GEO database. (**C, D**) The protein expression of PPP1R15A **(C)** and JUN **(D)** in our GC cohorts, calculating and comparing H-scores. (**E**, **F**) The prognostic value of PPP1R15A **(E)** and JUN **(F)** protein levels in our GC cohorts. **(G)** The prognostic value of PPP1R15A and JUN combination in our GC cohorts. **(H)** The multivariate Cox regression analyses of gene expressions presented by forest map.

The TCGA database and our GC cohort were used to illustrate the expression model and the protumor effects of PPP1R15A and JUN in GC. The PPP1R15A gene was highly expressed in cancer compared with normal tissues, combining the normal samples in GTEx with TCGA-STAD (Fig. 3A). Patients with high PPP1R15A expression demonstrated poorer overall survival among 875 patients with GC in the GEO database (Fig. 3B).

IHC staining was performed in 246 cancer and the matched adjacent control tissues in our GC cohort. H-Score was used for quantitative analysis according to the protein staining intensity and the percentage of each intensity. The results indicated both increased PPP1R15A and JUN expression levels in cancer tissues (Fig. 3C and D). The patients were categorized based on the median grouping method into low and high PPP1R15A/JUN expression groups. The results indicated that high PPP1R15A/JUN expression was associated with poor prognosis in patients with GC (*P* < 0.01) (Fig. 3E and F).

Furthermore, the risk of death increased in high PPP1R15A (hazard ratio [HR]: 1.58, 95% confidence interval [CI]: 1.14–2.20) and JUN expression groups (HR: 1.60, 95% CI: 1.15–2.21). The patients were categorized into four groups, considering the regulatory effects of JUN on PPP1R15A: [1] LL: low PPP1R15A and JUN expressions; [2] HL: high PPP1R15A and low JUN expressions; [3] HH: high PPP1R15A and JUN expressions; [4] LH: low PPP1R15A and high JUN expressions. Patients in other groups demonstrated poorer prognosis compared with the LL group (*P* < 0.001) (Fig. 3G). Additionally, the risk of death in the HL (HR: 2.22, 95% CI: 1.33–3.69), HH (HR: 2.32, 95% CI: 1.47–3.65), and LH groups were higher (HR: 2.21, 95% CI: 1.35–3.64) (Fig. 3H) compared with the LL group, indicating that aberrantly active expression of either JUN or PPP1R15A promoted GC development.

### The protumor role of PPP1R15A in GC in vitro and in vivo

**Fig. 4:**
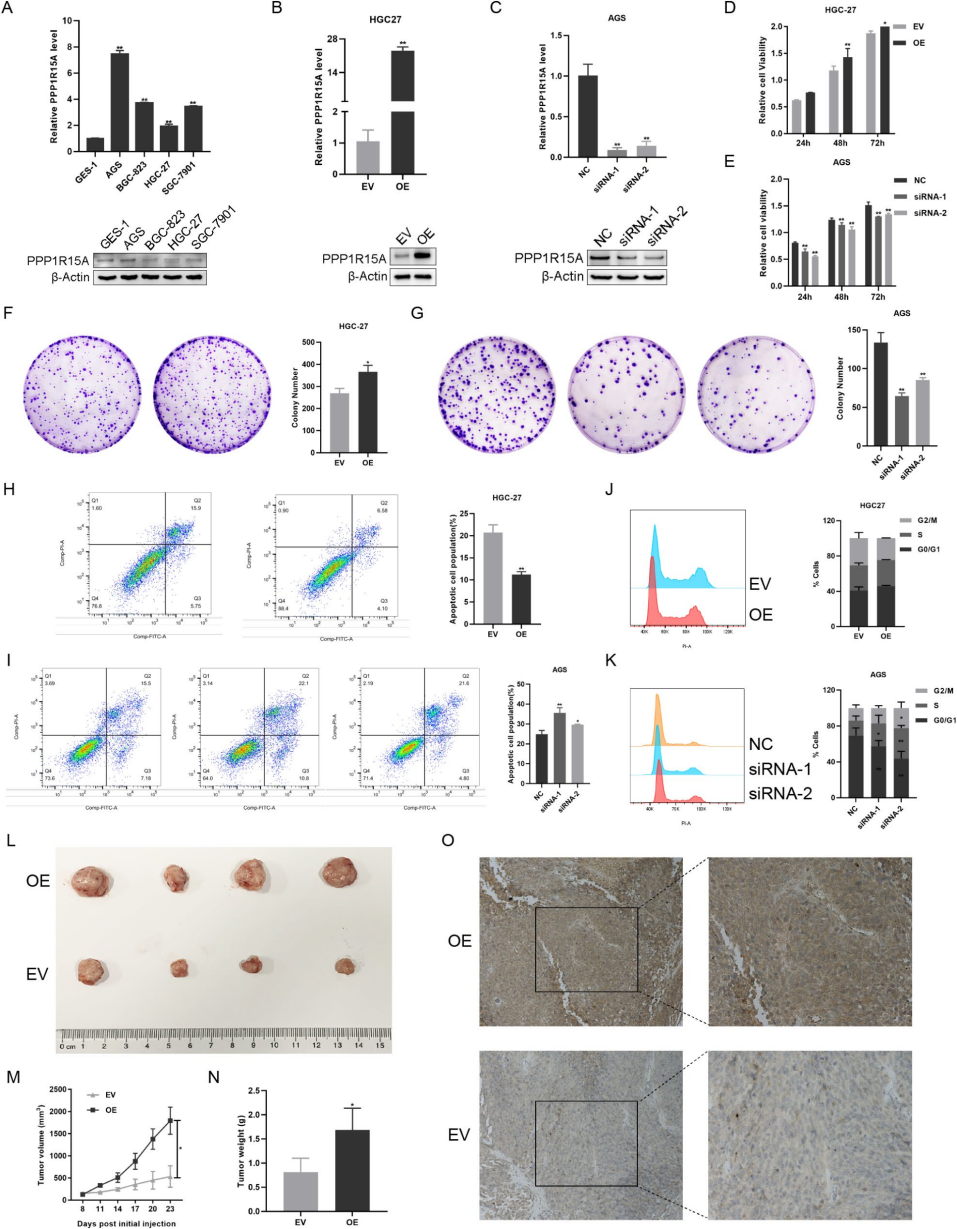
The pro-tumor role of PPP1R15A in GC in vitro and in vivo. **(A)** The mRNA and protein expression level of PPP1R15A in gastric mucosal epithelial cell line GES-1 and multiple GC cell lines. (**B**, **C**) The mRNA and protein expressions of PPP1R15A after overexpression vector transfection in HGC-27 **(B)** and siRNAs transfection in AGS **(C)**. (**D**, **E**) Cell proliferation ability after PPP1R15A overexpression **(D)** and knockdown **(E)**. (**F**, **G**) Colony formation ability of cells after PPP1R15A overexpression (**F**) and knockdown **(G)**. (**H**, **I**) Cell apoptosis after PPP1R15A overexpression **(H)** and knockdown **(I)**. (**J**, **K**) Cell cycle after PPP1R15A overexpression **(J)** and knockdown **(K)**. **(L)** Transplantation tumor model using HGC-27 with stable overexpressing PPP1R15A. **(M)** Subcutaneous tumor volume of nude mice. **(N)** Subcutaneous tumor weight of nude mice. (**O**) PPP1R15A protein expression in the subcutaneous tumor tissues of nude mice. EV: empty vector group; OE: overexpression vector group; NC: control group; siRNA: knockdown group

RT-qPCR and Western blot detected PPP1R15A expression in immortalized gastric mucosal epithelial cell line GES-1 and GC cell lines AGS, BGC-823, HGC-27, and SGC-7901. The mRNA of PPP1R15A in GC cell lines were overexpressed compared with GES-1 (Fig. 4A). HGC-27 and BGC-823 were then selected for PPP1R15A overexpression using plasmid (Fig. 4B, SFig. 5 A). AGS and SGC-7901 were selected for PPP1R15A knockdown using siRNA (siRNA-1 and siRNA-2) (Fig. 4C, SFig. 5B). CCK-8 test revealed increased cancer cell viability after PPP1R15A overexpression (Fig. 4D, SFig. 5 C), which decreased after PPP1R15A knockdown (Fig. 4E, SFig. 5D). Further colony formation assay indicated that the proliferation ability of cells was dependent on PPP1R15A expression (Fig. 4F–G, SFig. 5E–F).

The effects of PPP1R15A on apoptosis and cell cycle were then detected by flow cytometry. The apoptosis of cells was decreased by PPP1R15A overexpression (Fig. 4H, SFig. 5G), which increased after PPP1R15A knockdown (Fig. 4I, SFig. 5H). More GC cells were arrested in the G0/G1 phase after PPP1R15A overexpression (Fig. 4J, SFig. 5I) as well as in the G2/M phase after PPP1R15A knockdown (Fig. 4K, SFig. 5 J). Therefore, PPP1R15A inhibited cell apoptosis and induced G0/G1 arrest.

The lentivirus packaged with PPP1R15A overexpression vector was constructed to investigate the protumor role of PPP1R15A in GC in vivo, and HGC-27 with stable overexpressed PPP1R15A (OE group) was selected to develop the transplantation tumor model in nude mice, and the empty vector was established as control (EV group). The results revealed higher subcutaneous tumor volume and weight of the OE group than those of the EV group (Fig. 4L–N), indicating that PPP1R15A increases the tumorigenic ability of GC cells. Additionally, IHC images confirmed that PPP1R15A was overexpressed in the tumor tissue of the OE group (Fig. 4O).

### PPP1R15A induced cell autophagy in GC

HGC-27 with stable PPP1R15A overexpression and its control group were analyzed by RNA-seq and metabolite sequencing (Metabolite-seq) to illustrate the mechanisms of PPP1R15A. RNA-seq identified 6,857 differentially expressed genes, including 3,546 downregulated genes and 3,311 upregulated genes (*P* < 0.05) (SFig. 6 A). GO and KEGG enrichment analyses indicated that PPP1R15A regulated cell autophagy, lysosome organization, autophagosome maturation, and assembly (SFig. 6B-C). Meanwhile, 5,588 differential metabolites were identified by metabolite-seq, including 2,770 downregulated metabolites and 2,818 upregulated metabolites (*P* < 0.05) (SFig. 6D). Both primary and secondary metabolites enrichment analyses revealed that lysosome and autophagy belonging to cellular processing were regulated by PPP1R15A (SFig. 6E-F). Noteworthily, both RNA-seq and metabolite-seq preliminarily revealed that PPP1R15A regulates cell autophagy procedure.

PPP1R15A was highly correlated with autophagy pathway-related genes, ATG3, ATG5, BECN1, SQSTM1 (p62), ULK1, etc. based on the TCGA-STAD database (SFig. 6G). The patients were then categorized into low and high expression groups of PPP1R15A following the median grouping method, and the above genes were overexpressed in the high PPP1R15A expression group (SFig. 6 H). GO and KEGG pathway enrichment analyses indicated that PPP1R15A was associated with autophagy, macroautophagy, lysosome, and cell response to starvation (SFig. 6I-J).

**Fig. 5:**
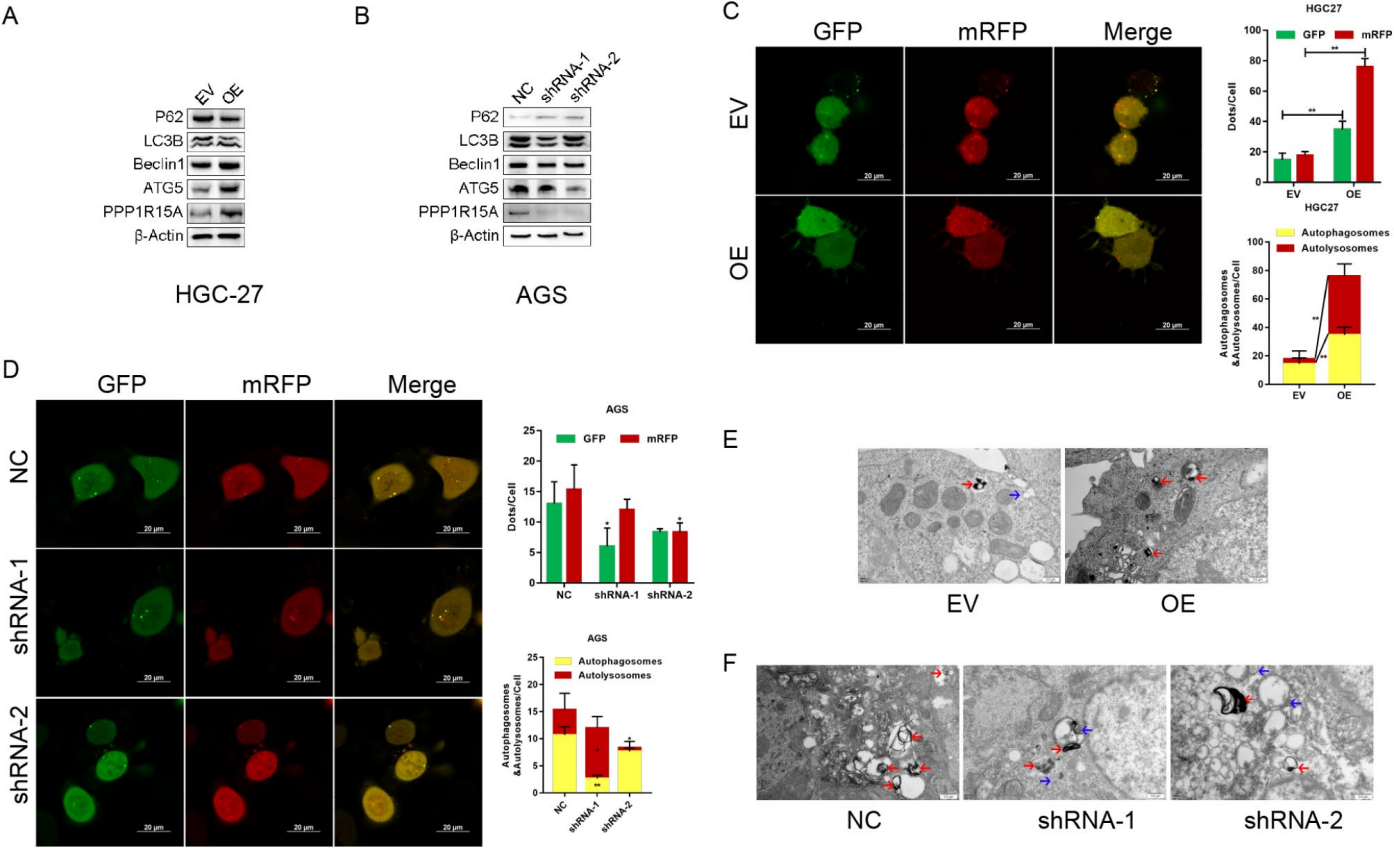
PPP1R15A promoted cell autophagy in GC. (**A**, **B**) Effects of PPP1R15A overexpression in HGC-27 **(A)** and knockdown in AGS **(B)** on autophagy-related protein expressions. (**C**, **D**) Effects of PPP1R15A overexpression in HGC-27 **(C)** and knockdown in AGS **(D)** on cell autophagy observed by laser scanning confocal microscope. The red spots indicated autolysosomes, whereas the yellow spots denoted autophagosomes. (**E**, **F**) Effects of PPP1R15A overexpression in HGC-27 **(E)** and knockdown in AGS **(F)** on autophagosomes and autolysosomes observed by the transmission electron microscopy. The blue arrows indicated autophagosomes and autophagy prophase, whereas the red arrows represented autolysosomes. EV: empty vector group; OE: overexpression vector group; NC: control group; shRNA: knockdown group

WB was used to detect the expression of autophagy-related proteins to verify the regulation effects of PPP1R15A on cell autophagy, revealing that PPP1R15A overexpression increased Beclin1 and ATG5 expressions, decreased p62 expression, and promoted LC3B cleavage in HGC-27 and BGC-823 (Fig. 5A, SFig. 7 A), whereas PPP1R15A knockdown downregulated Beclin1, ATG5 expression, upregulated p62 expression, and inhibited LC3B cleavage in AGS and SGC-7901 (Fig. 5B, SFig. 7B). Meanwhile, the cells were infected with mRFP-GFP-LC3B adenovirus to track the autophagy flow. The yellow spots (created by the overlap of red and green fluorescence) represent autophagosomes, whereas the red spots represent autolysosomes. The laser scanning confocal microscope results indicated that PPP1R15A overexpression promoted autophagosome and autolysosome formation in HGC-27 and BGC-823 (Fig. 5C, SFig. 7 C), whereas PPP1R15A knockdown inhibited the autophagy flow of AGS and SGC-7901 (Fig. 5D, SFig. 7D). Additionally, transmission electron microscopy was performed to understand the effects of PPP1R15A on autophagy. The blue arrows represent autophagosomes and autophagy prophase, and the red arrows represent autolysosomes. The results revealed that PPP1R15A overexpression promoted the assembly and maturation of autophagosomes and autolysosomes in HGC-27 (Fig. 5E), whereas PPP1R15A knockdown inhibits the autophagy of AGS (Fig. 5F). The above results indicated that PPP1R15A promoted cell autophagy of GC cells.

### PPP1R15A was essential for GC cells to adapt to energy stress

**Fig. 6:**
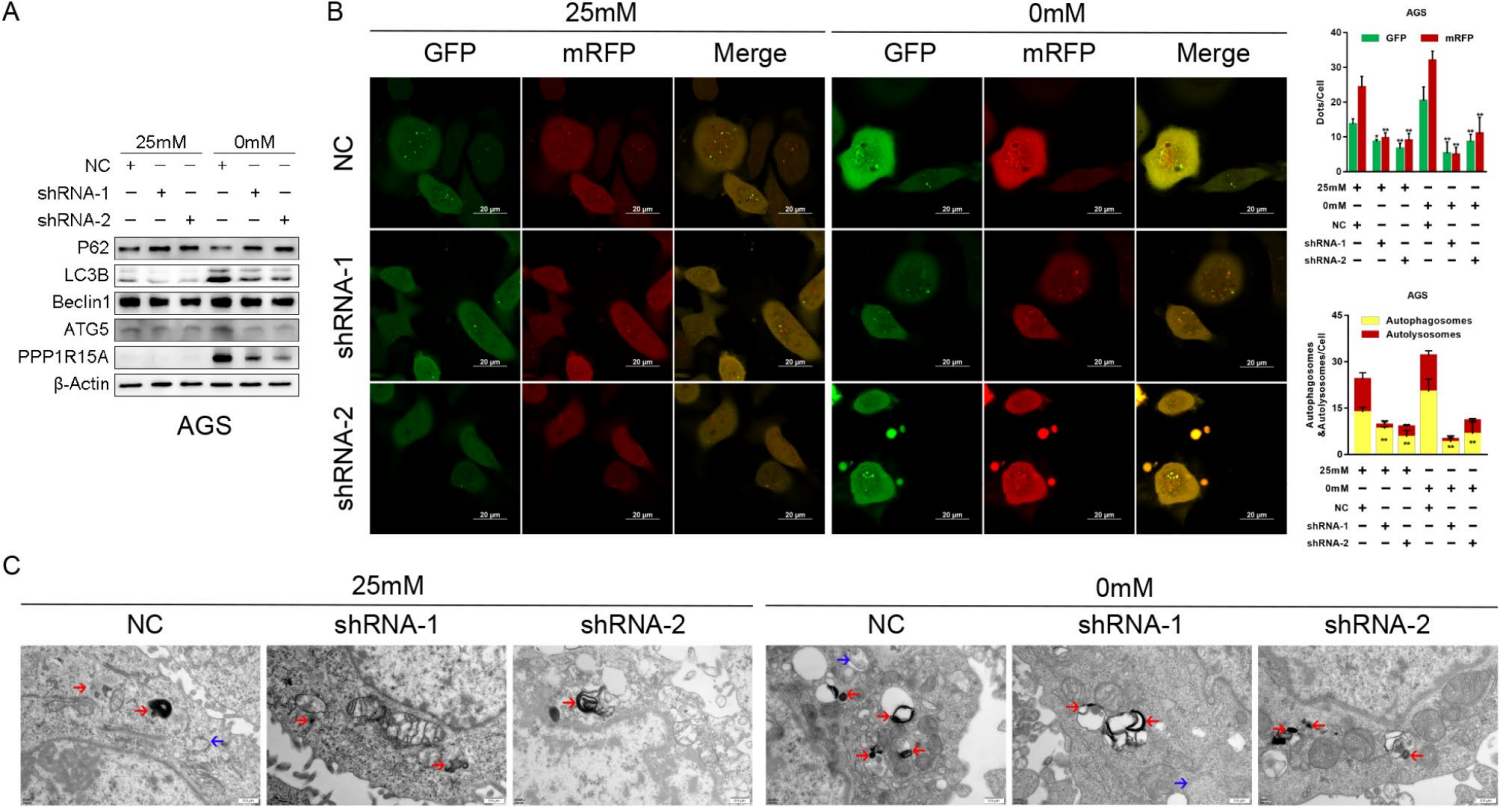
PPP1R15A promoted cell autophagy to adapt energy stress in GC. **(A)** Effects of PPP1R15A knockdown on autophagy-related protein expressions in AGS after glucose deprivation for 24 h. **(B)** Effects of PPP1R15A knockdown on cell autophagy after glucose deprivation for 24 h in AGS observed by laser scanning confocal microscope. The red spots denoted autolysosomes, whereas the yellow spots indicated autophagosomes. **(C)** Effects of PPP1R15A knockdown on autophagosomes and autolysosomes in AGS observed by transmission electron microscopy. The blue arrows represented autophagosomes and autophagy prophase, whereas the red arrows indicated autolysosomes

We simulated glucose deprivation in AGS and SGC-7901 which had stable knockdown of PPP1R15A to understand the function of PPP1R15A under energy stress. Similar to Figs. 1E and 2E, energy stress-activated PPP1R15A, Beclin1, and ATG5 expression, downregulated p62 expression, and induced LC3B cleavage. However, PPP1R15A knockdown reversed the effects of energy stress on autophagy-related proteins both in AGS and SGC-7901 (Fig. 6A, SFig. 8 A). Further mRFP-GFP-LC3B adenovirus infection confirmed that energy stress-induced autophagosome and autolysosome formation, whereas PPP1R15A knockdown inhibited the density of green and red fluorescence which activated by energy stress (Fig. 6B, SFig. 8B), indicating that PPP1R15A knockdown blocked the autophagy procedure in different extents. Additionally, transmission electron microscopy revealed that glucose deprivation promoted the assembly and maturation of autophagosomes and autolysosomes, whereas PPP1R15A knockdown significantly inhibited the process (Fig. 6C).

## Discussion

Glucose deprivation is a ubiquitous condition in the tumor microenvironment, and it inhibits GC cell proliferation, promotes apoptosis, and causes G1/S cell cycle arrest in our present study, indicating the negative effect of energy stress on GC progression. However, emerging shreds of evidence revealed that cancer cells can evolve to adapt the harsh environments. AMPK-dependent activation of P53 promotes cell cycle arrest in the G1/S phase, thereby improving cell survival during glucose starvation [14]. P53 promoted P21-dependent cell cycle arrest and antioxidant biosynthesis in serine-depleted cells, which together supported environmental adaptation and cell survival [15]. Trx-1 [16] and RPIA [17] support ROS clearance by producing NADPH and promoting cell survival under energy stress. DLC3/MACC1 axis regulated GC cell chemotaxis to escape glucose deprivation [18].

Autophagy is crucial for cells to maintain balance and dysfunction, and autophagy is associated with the pathogenesis of various diseases, including cancer, neurodegenerative disorders, immune system anomalies, etc [19]. Autophagy plays different roles in various tumor development stages and may promote or inhibit tumor progression; thus, most clinical measures to intentionally interfere with autophagy have been implemented in cancer treatment [20]. Additionally, autophagy enables cells to adapt to various environmental and endogenous pressures, including hypoxia, reactive oxygen species, and cell damage [21]. Autophagy endows cells with more survival advantages under nutrient restriction conditions from the perspective of evolution [10–12].

Hypoxia induces downstream gene expression by activating AMPK, HIF-1α, or ATF4, thereby mediating autophagy to promote cell survival [22]. Oxidative stress triggers autophagy via either NF-kB or LKB1-AMPK pathways. In contrast, activated autophagy promotes antioxidant responses through the Keap1-Nrf2 pathway, thereby reducing oxidative stress [23, 24]. Metabolic stress activates mTOR and AMPK pathways to increase survival by activating autophagy [25]. Notably, our sequencing data observed most of the above pathways or molecules, indicating that cells adapt to various environmental stresses through similar mechanisms.

Glucose deprivation activated intracellular autophagic activity via multiple pathways [26]. Mec1 regulated glucose starvation-induced autophagy by controlling the PAS recruitment of Atg13 in yeast [27]. ULK1-mediated PIKfyve activation improved autophagy flux upon glucose starvation in diverse cell lines [28]. Nrf2 via its antioxidant activity protects breast cancer cells during glucose deprivation-induced autophagy [29]. SIRT1-FoxO1-Rab7 increases autophagy in GC cells upon glucose starvation [30]. However, the activity and mechanism of autophagy to help GC resist the effects of energy stress remained unclear.

PPP1R15A is a protein that is induced by multiple stressors, including DNA damage, heat shock, nutritional deficiency, energy depletion, and endoplasmic reticulum stress [31]. The function of PPP1R15A has remained controversial. Some studies revealed that it may induce apoptosis [32], whereas other studies demonstrated that it could prevent apoptosis or tissue injury [33, 34]. PPP1R15A, also known as growth arrest and DNA damage-inducible protein (GADD34), dephosphorylate eukaryotic translation initiation factor 2α (eIF2α), which reverse the protein synthesis shutdown induced by eIF2α phosphorylation under stress conditions, thereby preventing cell death [35, 36]. Additionally, PPP1R15A inhibited the mTOR signaling pathway [37] to protect cells from glucose depletion-induced apoptosis [38]. PPP1R15A attenuates LPS-induced acute liver injury by inhibiting macrophage activation [39]. PPP1R15A positively regulated MCL-1 expression in liver cancer cells, which is an essential survival factor in many cancers [34]. In vivo and in vitro experiments revealed that PPP1R15A promotes the proliferation and tumorigenic ability, G1/S cell cycle arrest, and inhibits apoptosis of GC cells in the present study. TCGA data and our GC cohort indicated that patients with high PPP1R15A expression exhibited a poor prognosis, indicating that GC cells upregulated PPP1R15A expression under energy stress to alleviate the challenges they were facing.

**Fig. 7:**
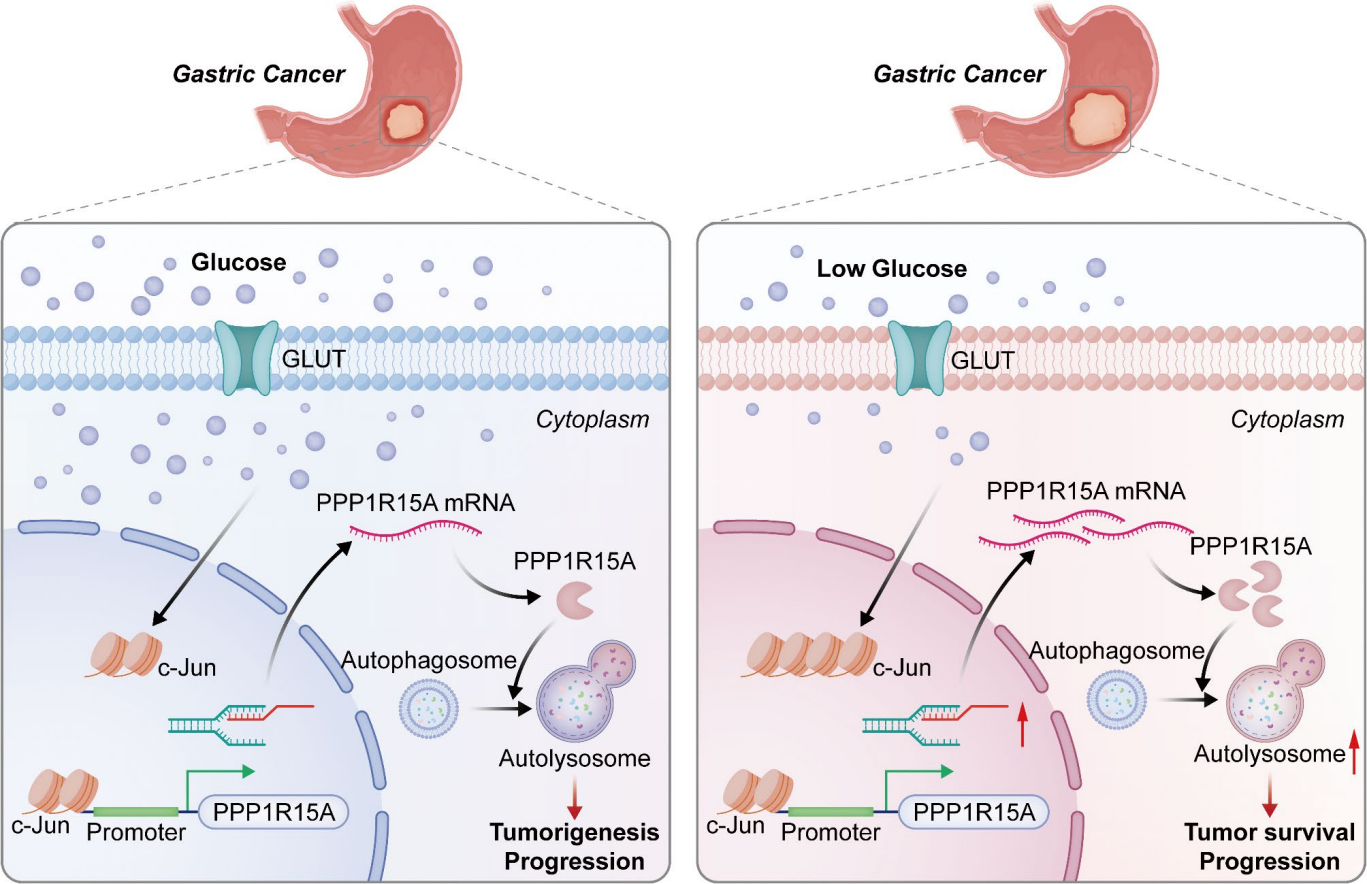
The scheme of the study. High c-Jun induced by energy stress promotes the transcription of PPP1R15A in GC cells, which improves autophagy by promoting the biosynthesis and maturation of autophagosomes and contributes to GC tumorigenesis and progression

PPP1R15A was once associated with the autophagy pathway in the rat tendon injury model [40]. Furthermore, PPP1R15A may be a modulator of autophagy during starvation which could enable lysosomal biogenesis and sustain autophagy flux by regulating translation [36] or suppressing the mTOR pathway [37]. Macrophages may prevent excessive proliferation and facilitate the use of degraded amino acids by increasing PPP1R15A expression when exposed to LPS [41]. The present study indicated that energy stress-induced the biosynthesis, assembly, and maturation of autophagosomes and autolysosomes, and PPP1R15A knockdown exerts an inhibitory effect on the energy stress-activated cellular autophagy, indicating that PPP1R15A plays a crucial role in GC cell survival by regulating cellular autophagy under energy stress (Fig. 7).

C-Jun is a member of the JUN family of transcription factors and c-Jun overexpression significantly affects a range of biological processes, including apoptosis, proliferation, invasion, and migration [42, 43]. Both JUN and PPP1R15A expression levels were higher in GC tissues in our GC cohort than in adjacent control tissues, and high expression was associated with poor prognosis. The combined analysis results of JUN and PPP1R15A revealed that the prognosis of patients with low expression of both genes was better than that of other groups. JUN and PPP1R15A were all independent risk factors for GC prognosis. Both genes play an important role in tumor progression. Additionally, c-Jun was significantly associated with lymph node metastasis in oral squamous cell carcinoma [44]. SNP polymorphism PPP1R15A was significantly associated with GC, nasopharyngeal carcinoma, and lymphoma [45]. Increased PPP1R15A expression was related to poor prognosis in patients with thyroid cancer [46]. Targeting PPP1R15A activity inhibits tumor growth [47].

## Conclusions

We simulated the adverse environment encountered during tumor development and investigated the coping mechanisms of tumor cells by developing an energy stress model. The results revealed that PPP1R15A affected GC cell survival by regulating autophagy under energy stress. Both PPP1R15A and autophagy may be therapeutic targets for GC. Exploring the survival mechanism of cancer cells in a harsh environment is crucial. These adaptive mechanisms collectively enable tumor cells to survive and proliferate, thereby contributing to tumor progression and even therapy resistance.

## Electronic supplementary material

Below is the link to the electronic supplementary material.

Supplementary Material 1

Supplementary Material 2

Supplementary Material 3

Supplementary Material 4

Supplementary Material 5

Supplementary Material 6

Supplementary Material 7

Supplementary Material 8

Supplementary Material 9

Supplementary Material 10

Supplementary Material 11

## Data Availability

The data that support the findings of this study are available from the corresponding author upon reasonable request.

## Abbreviations

GC: Gastric cancer
PPP1R15A: Protein phosphatase 1 regulatory subunit 15 A
DEG: Differentially expressed genes
GADD34: Growth arrest and DNA damage-inducible protein
eIF2α: Eukaryotic translation initiation factor 2α
HK1: Glucose-6-phosphate dehydrogenase
LDHA: Lactate dehydrogenase
PDK1: Pyruvate dehydrogenase kinase 1
GLUT1: Glucose transporter member 1
RT-qPCR: Quantitative real-time polymerase chain reaction
IHC: Immunohistochemistry
WB: Western blot
ChIP: Chromatin immunoprecipitation
TCGA: The Cancer Genome Atlas
GEO: Gene Expression Omnibus
GTEx: Genotype-Tissue Expression
GO: Gene ontology
KEGG: Kyoto encyclopedia of genes and genomes
GSEA: Gene set enrichment analysis
