# Supplementary figures and images for: Protein phosphatase 1 regulatory subunit 15 A (PPP1R15A) promoted the progression of gastric cancer by activating cell autophagy under energy stress
Source: J Exp Clin Cancer Res. 2025 Feb 13;44:52. doi: 10.1186/s13046-025-03320-y (PMC11823012; doi:10.1186/s13046-025-03320-y)

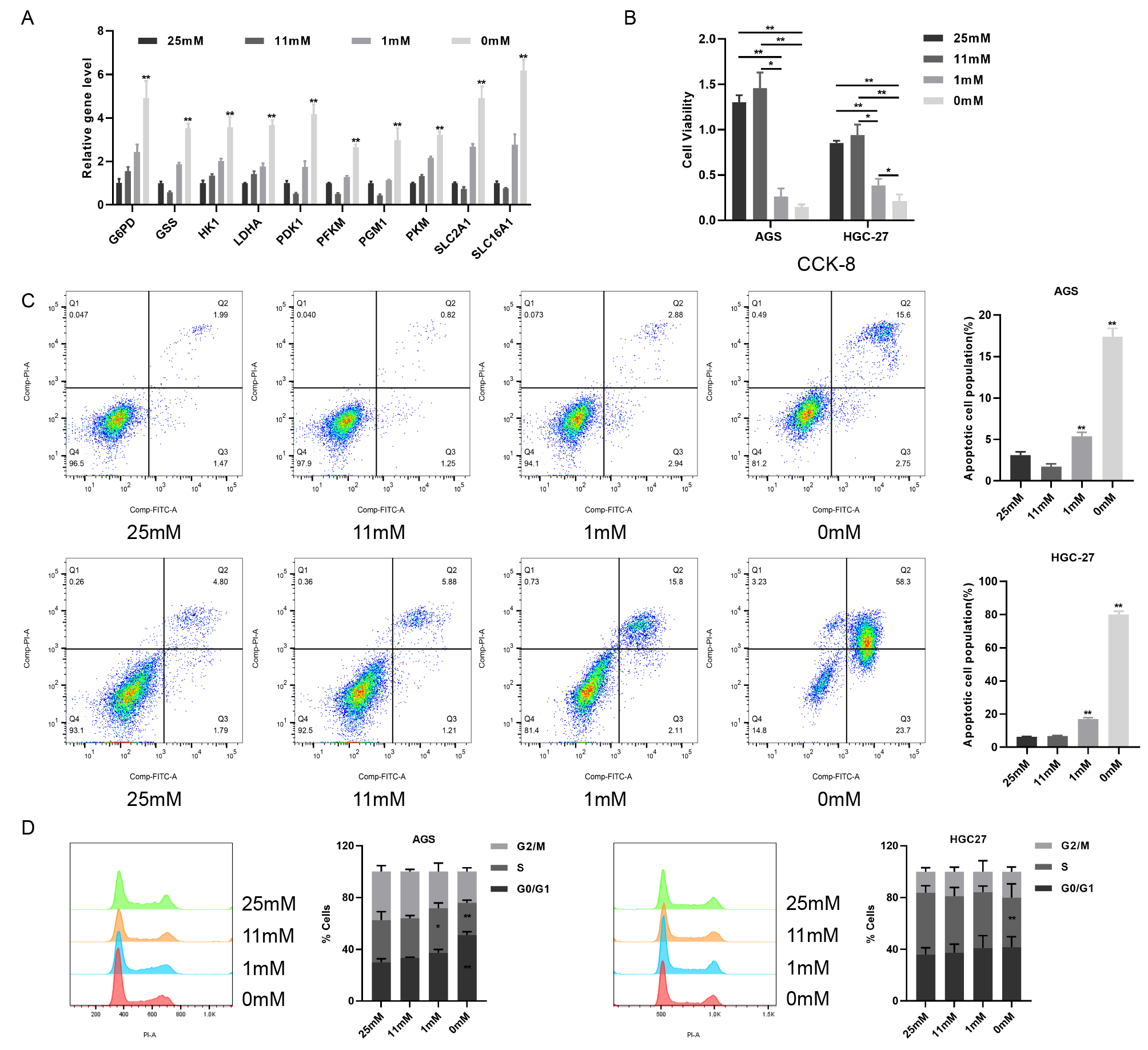

Supplement: Supplementary file 1 — Supplementary Material 1 [file 13046_2025_3320_MOESM1_ESM.png]

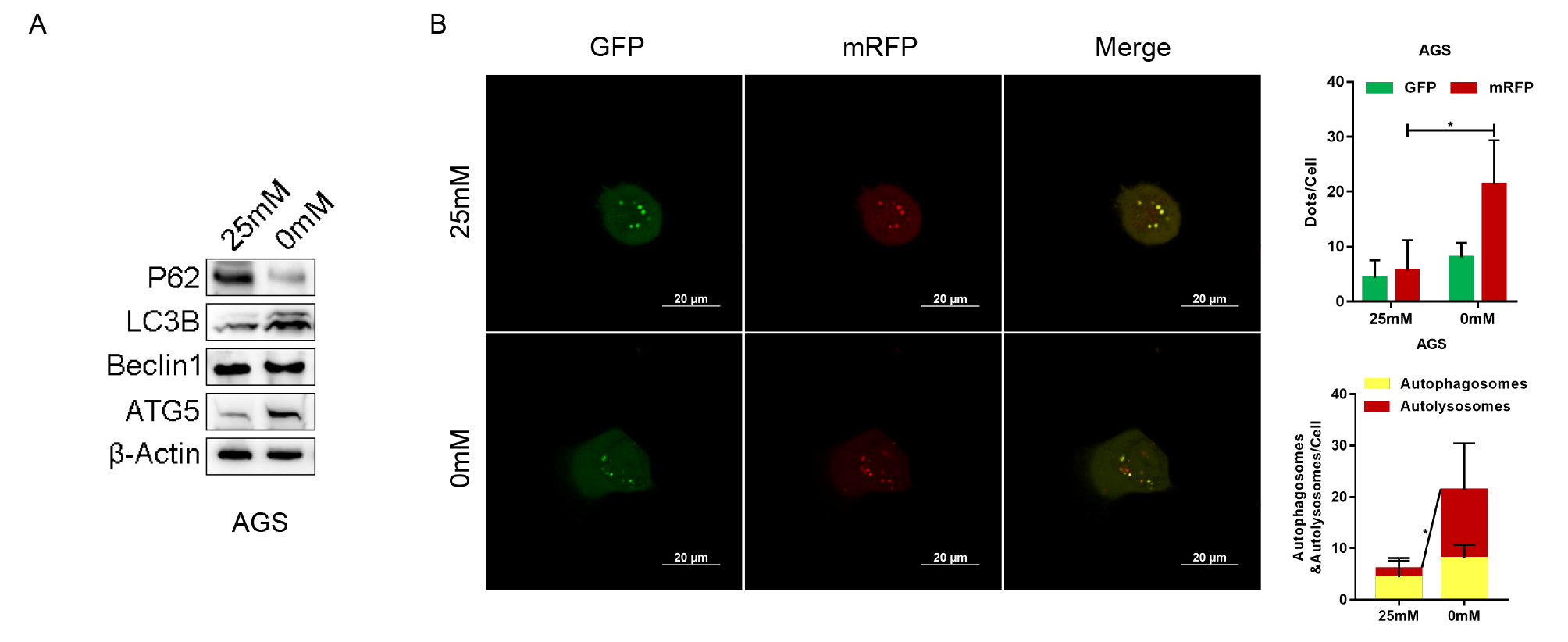

Supplement: Supplementary file 2 — Supplementary Material 2 [file 13046_2025_3320_MOESM2_ESM.png]

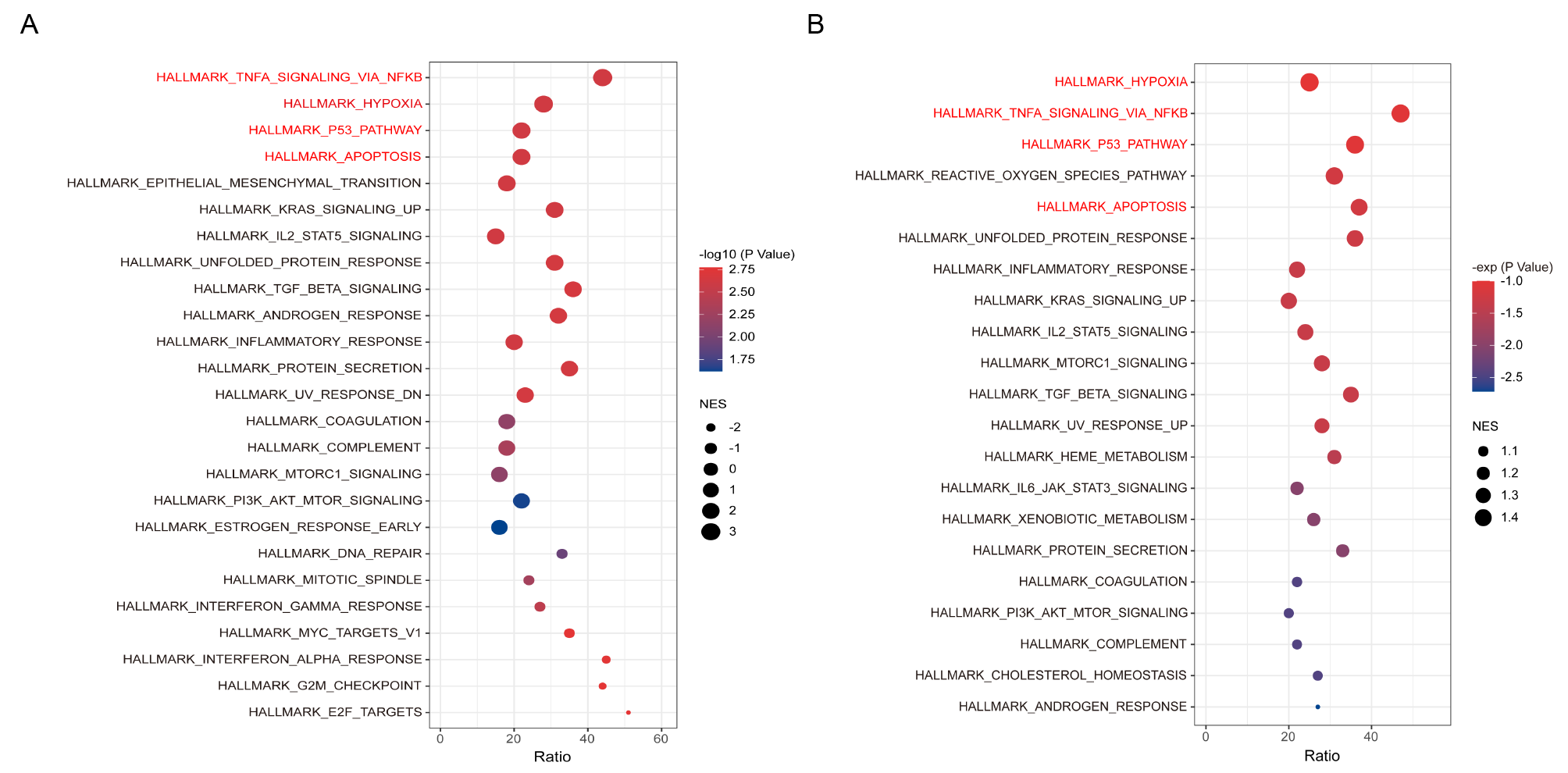

Supplement: Supplementary file 3 — Supplementary Material 3 [file 13046_2025_3320_MOESM3_ESM.png]

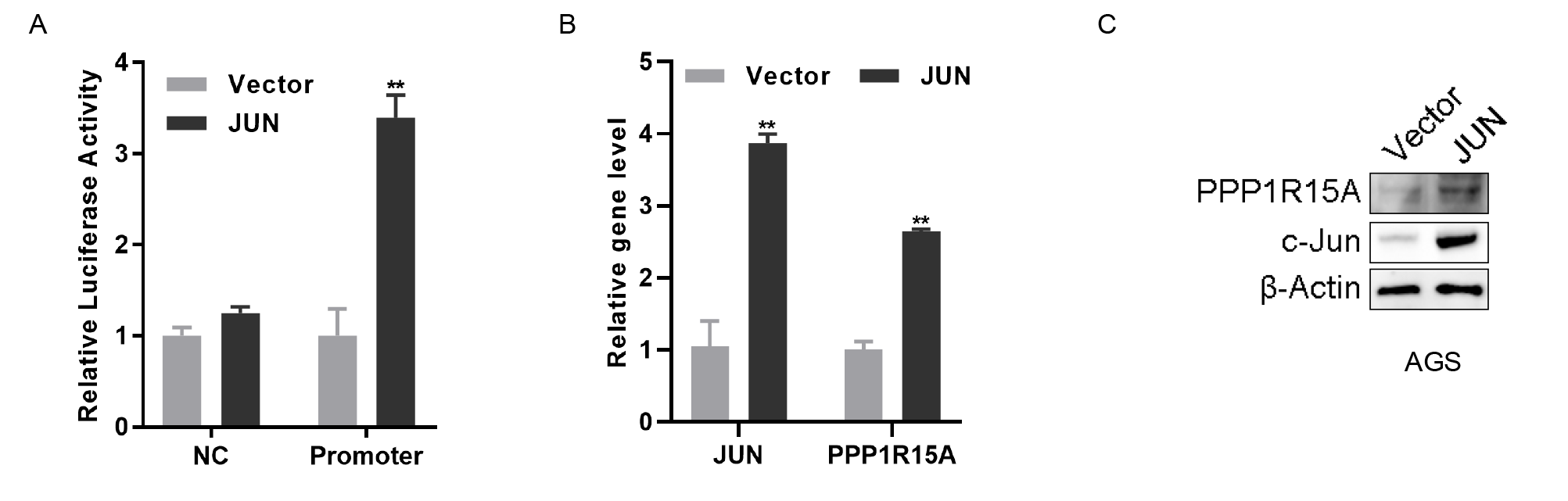

Supplement: Supplementary file 4 — Supplementary Material 4 [file 13046_2025_3320_MOESM4_ESM.png]

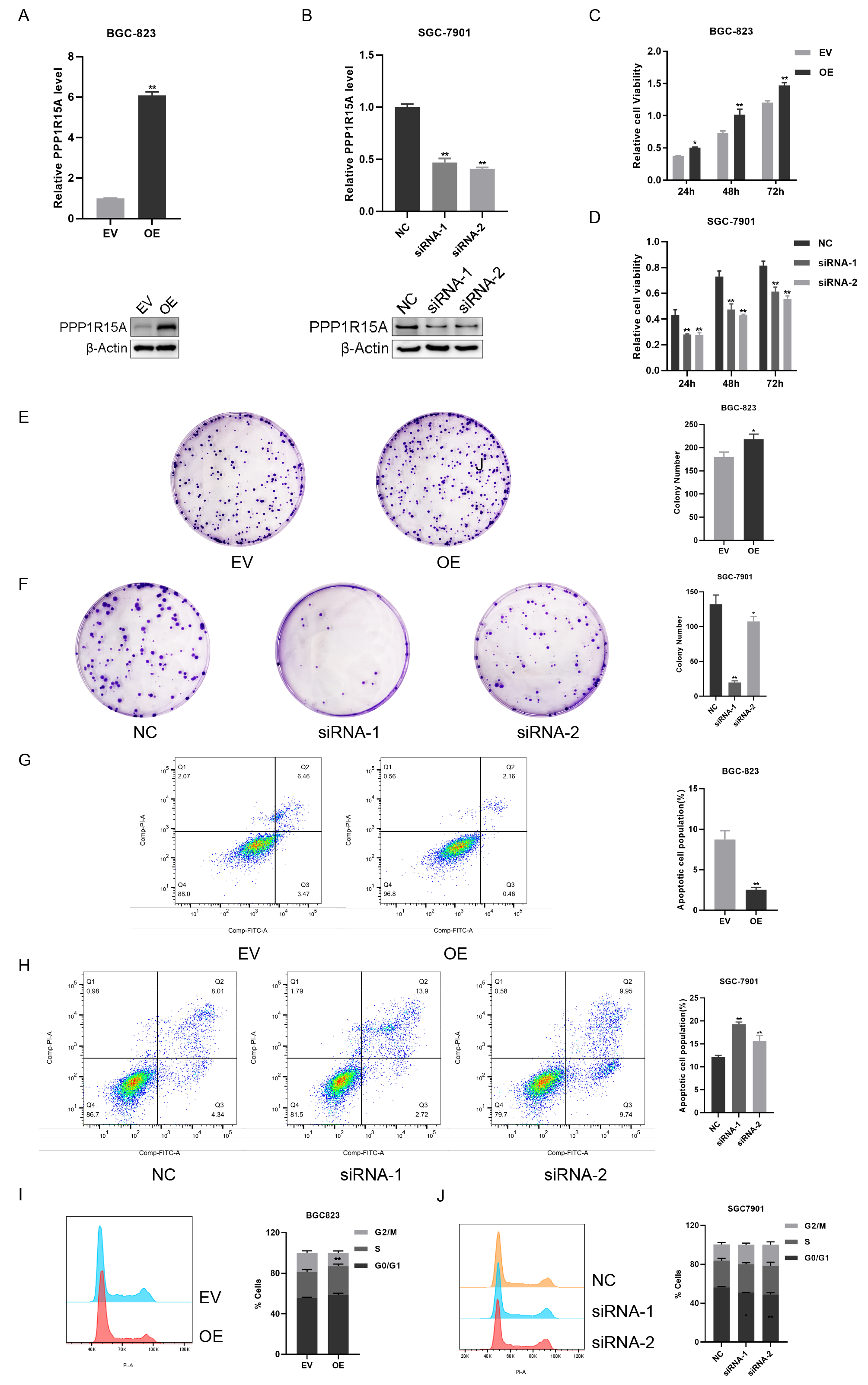

Supplement: Supplementary file 5 — Supplementary Material 5 [file 13046_2025_3320_MOESM5_ESM.png]

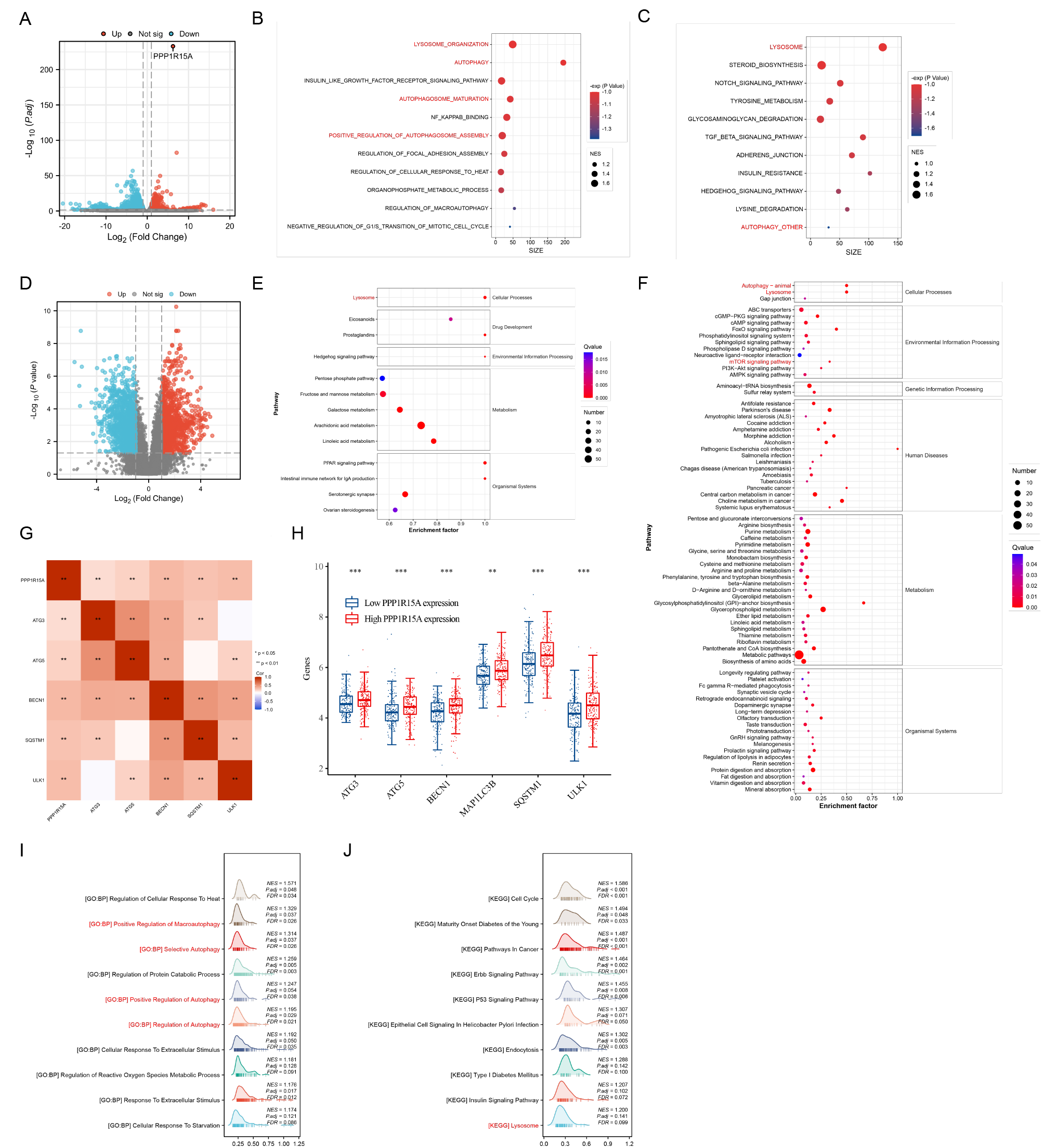

Supplement: Supplementary file 6 — Supplementary Material 6 [file 13046_2025_3320_MOESM6_ESM.png]

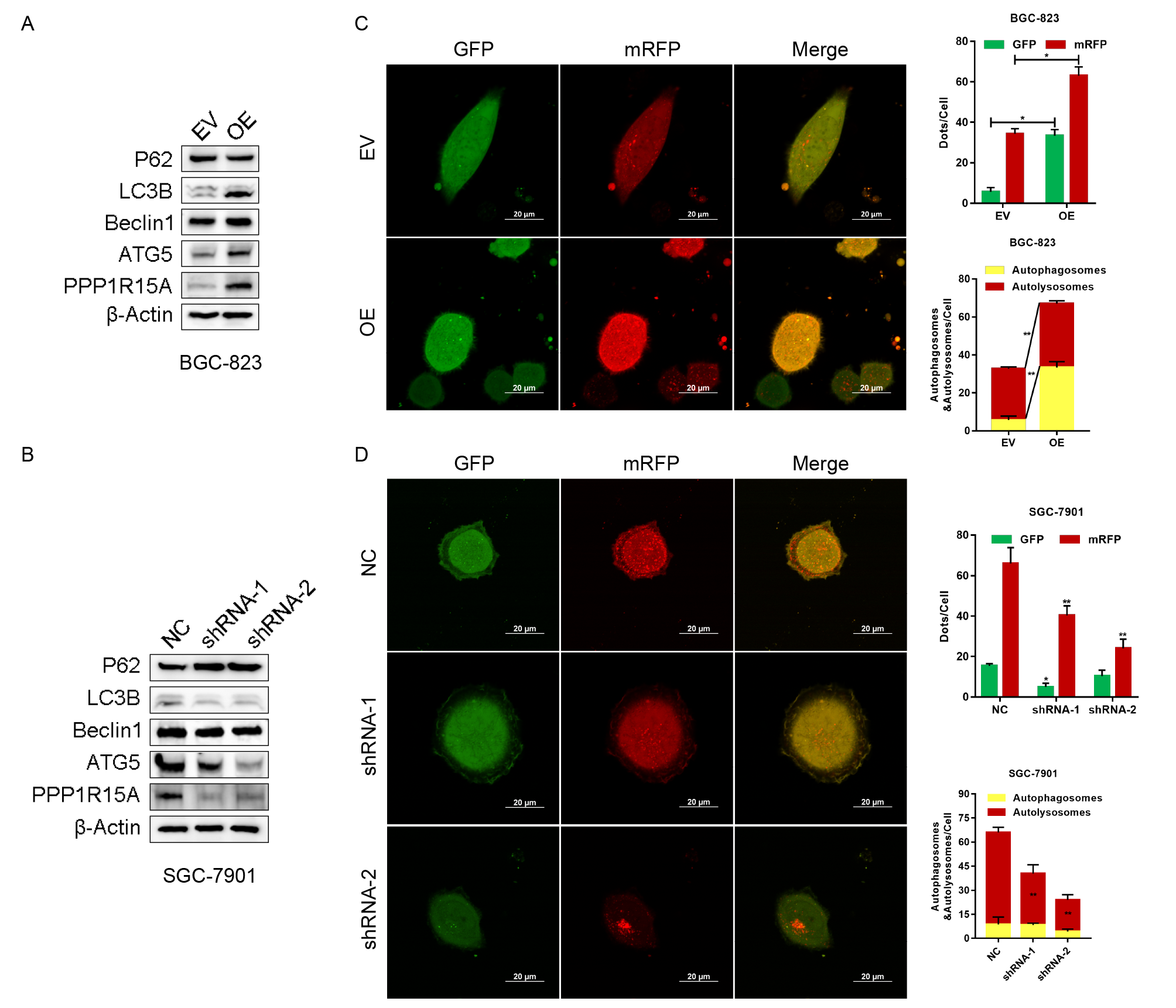

Supplement: Supplementary file 7 — Supplementary Material 7 [file 13046_2025_3320_MOESM7_ESM.png]

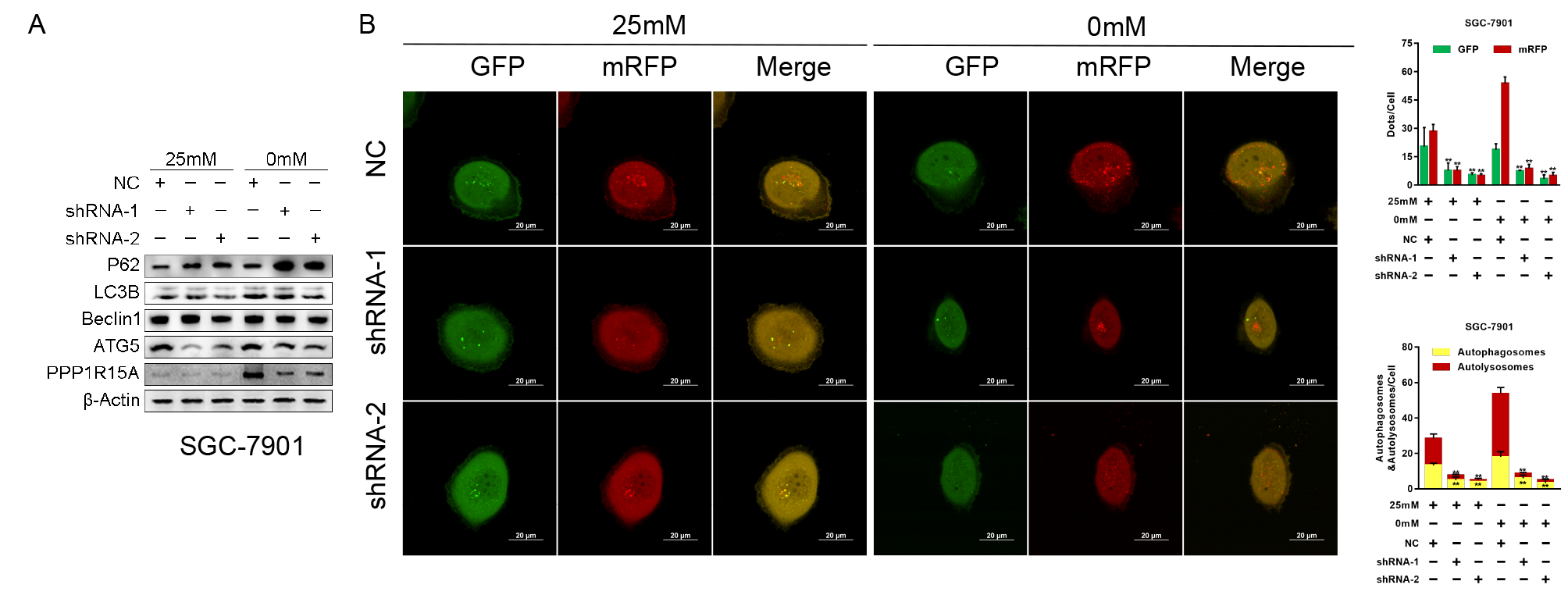

Supplement: Supplementary file 8 — Supplementary Material 8 [file 13046_2025_3320_MOESM8_ESM.png]
