# Supplementary material for: Protein phosphatase 1 regulatory subunit 15 A (PPP1R15A) promoted the progression of gastric cancer by activating cell autophagy under energy stress
Source: J Exp Clin Cancer Res. 2025 Feb 13;44:52. doi: 10.1186/s13046-025-03320-y (PMC11823012; doi:10.1186/s13046-025-03320-y)
Supplement: Supplementary file 10 — Supplementary Material 10 [file 13046_2025_3320_MOESM10_ESM.docx]

Supplementary Table 1 Characteristics of patients in the GC cohort

| Variable | Classification | Counts n (%) |
| --- | --- | --- |
| Age (year) | <65 | 149 (60.6%) |
|  | ≥65 | 97 (39.4%) |
| Gender | Male | 187 (76%) |
|  | Female | 59 (24%) |
| Vascular invasion | No | 53 (21.5%) |
|  | Yes | 193 (78.5%) |
| Neural invasion | No | 75 (30.5%) |
|  | Yes | 171 (69.5%) |
| Chemotherapy | No | 125 (50.8%) |
|  | Yes | 121 (49.2%) |
| M stage | M0 | 222 (90.2%) |
|  | M1 | 24 (9.8%) |
| T stage | T1 | 6 (2.4%) |
|  | T2 | 11 (4.5%) |
|  | T3 | 125 (50.8%) |
|  | T4 | 104 (42.3%) |
| N stage | N0 | 46 (18.7%) |
|  | N1 | 34 (13.8%) |
|  | N2 | 69 (28%) |
|  | N3 | 97 (39.4%) |
| TNM stage | I&II | 48 (19.5%) |
|  | III&IV | 198 (80.5%) |
| Tumor size (cm) | ≤5 | 108 (43.9%) |
|  | >5 | 138 (56.1%) |
| WHO classification | Adenocarcinoma | 184 (74.8%) |
|  | Mucinous adenocarcinoma | 33 (13.4%) |
|  | Signet ring cell carcinoma | 28 (11.4%) |
|  | Other | 1 (0.4%) |
| Histological grade | High | 47 (19.1%) |
|  | Low | 199 (80.9%) |
| Final mortality status | Alive | 86 (35.0%) |
|  | Dead | 160 (65.0%) |

Supplementary Table 2 The siRNA sequences for PPP1R15A

| RNA oligo |  | Sequence |
| --- | --- | --- |
| Negative control | Sense | 5’- UUCUCCGAACGUGUCACGUTT -3’ |
|  | Antisense | 5’- ACGUGACACGUUCGGAGAATT -3’ |
| siRNA-1 | Sense | 5’- GGAGGAGGAAGAUGAGGAUTT-3’ |
|  | Antisense | 5’- AUCCUCAUCUUCCUCCUCCTT-3’ |
| siRNA-2 | Sense | 5’- GGAAGAGGGAGUUGCUGAATT-3’ |
|  | Antisense | 5’- UUCAGCAACUCCCUCUUCCTT-3’ |

Supplementary Table 3 The shRNA sequences for PPP1R15A

| Name | Sequence |
| --- | --- |
| Negative control | 5’-TTCTCCGAACGTGTCACGT-3’ |
| shRNA-1 | 5’-GGAGGAGGAAGATGAGGAT-3’ |
| shRNA-2 | 5’-GGAAGAGGGAGTTGCTGAA-3’ |

Supplementary Table 4 The RT-qPCR primers

| Gene | Primer | Senquence |
| --- | --- | --- |
| β-actin | Forward | 5’-CATGTACGTTGCTATCCAGGC-3’ |
|  | Reverse | 5’-CTCCTTAATGTCACGCACGAT-3’ |
| PPP1R15A | Forward | 5’-GAATCAAGCCACGGAGGATA-3’ |
|  | Reverse | 5’-CAGGGAGGACACTCAGCTTC-3’ |
| JUN | Forward | 5’-TCCAAGTGCCGAAAAAGGAAG-3’ |
|  | Reverse | 5’-CGAGTTCTGAGCTTTCAAGGT-3’ |
| ATG5 | Forward | 5’-TTTGCATCACCTCTGCTTTC-3’ |
|  | Reverse | 5’-TAGGCCAAAGGTTTCAGCTT-3’ |
| BECN1 | Forward | 5’-GGTGTCTCTCGCAGATTCATC-3’ |
|  | Reverse | 5’-TCAGTCTTCGGCTGAGGTTCT-3’ |
| MAP1LC3B | Forward | 5’-AAGGCGCTTACAGCTCAATG-3’ |
|  | Reverse | 5’-CTGGGAGGCATAGACCATGT-3’ |
| SQSTM1 | Forward | 5’-GCACCCCAATGTGATCTGC-3’ |
|  | Reverse | 5’-CGCTACACAAGTCGTAGTCTGG-3’ |
| G6PD | Forward | 5’-ACCGCATCGACCACTACCT-3’ |
|  | Reverse | 5’-TGGGGCCGAAGATCCTGTT-3’ |
| GSS | Forward | 5’-GGAACATCCATGTGATCCGAC-3’ |
|  | Reverse | 5’-GCCATCCCGGAAGTAAACCA-3’ |
| HK1 | Forward | 5’-GCTCTCCGATGAAACTCTCATAG-3’ |
|  | Reverse | 5’-GGACCTTACGAATGTTGGCAA-3’ |
| LDHA | Forward | 5’-ATGGCAACTCTAAAGGATCAGC-3’ |
|  | Reverse | 5’-CCAACCCCAACAACTGTAATCT-3’ |
| PDK1 | Forward | 5’-GGATTGCCCATATCACGTCTTT-3’ |
|  | Reverse | 5’-TCCCGTAACCCTCTAGGGAATA-3’ |
| PFKM | Forward | 5’-CAAGGAACAGTGGTGGCT-3’ |
|  | Reverse | 5’-GAGGTTTAGACGGCAGCTT-3’ |
| PGM1 | Forward | 5’-AGGATGTGGCAATGAAATG-3’ |
|  | Reverse | 5’-CGCAAAACAAGGCAGAA-3’ |
| PKM | Forward | 5’-CATAGTGAAGCCGGGACTG-3’ |
|  | Reverse | 5’-GCTGTGATGGGTGGTGAA-3’ |
| SLC2A1 | Forward | 5’-ATTGGCTCCGGTATCGTCAAC-3’ |
|  | Reverse | 5’-GCTCAGATAGGACATCCAGGGTA-3’ |
| SLC16A1 | Forward | 5’-AGGTCCAGTTGGATACACCCC-3’ |
|  | Reverse | 5’-GCATAAGAGAAGCCGATGGAAAT-3’ |

Supplementary Table 5 The ChIP-PCR primers

| Gene | Primer | Sequence |
| --- | --- | --- |
| PPP1R15A-1 | Forward | 5’-GGGCCACGCATTTGATTGAC-3’ |
|  | Reverse | 5’-CAGGACTTCTCGCGGAGATT-3’ |
| PPP1R15A-2 | Forward | 5’-AAGGCATTGACCCTGAGCTG-3’ |
|  | Reverse | 5’-CTGACGTCACGAAGAGAGGC-3’ |

Supplementary Table 6 The antibodies for Western blot and IHC

| Antibody | Reagent merchant | Country |
| --- | --- | --- |
| Anti-ATG5 | Absin | China |
| Anti-Beclin1 | Abcam | UK |
| Anti-P62 | Absin | China |
| Anti-LC3B | Abcam | UK |
| Anti-c-Jun | Abcam | UK |
| Anti-PPP1R15A | Proteintech | USA |
| Anti-β-actin | Absin | China |
| Second Antibody | Absin | China |

Supplementary Table 7 Characteristics of patients in TCGA

| Variable | Classification | Counts n (%) |
| --- | --- | --- |
| Age (year) | < 65 | 155 (41.8%) |
|  | > = 65 | 216 (58.2%) |
| Gender | Female | 134 (35.7%) |
|  | Male | 241 (64.3%) |
| T stage | T1 | 19 (5.2%) |
|  | T2 | 80 (21.8%) |
|  | T3 | 168 (45.8%) |
|  | T4 | 100 (27.2%) |
| N stage | N0 | 111 (31.1%) |
|  | N1 | 97 (27.2%) |
|  | N2 | 75 (21%) |
|  | N3 | 74 (20.7%) |
| M stage | M0 | 330 (93%) |
|  | M1 | 25 (7%) |
| TNM stage | I | 53 (15.1%) |
|  | II | 111 (31.5%) |
|  | III | 150 (42.6%) |
|  | IV | 38 (10.8%) |
| Final mortality status | Alive | 228 (60.8%) |
|  | Dead | 147 (39.2%) |
