# Supplementary material for: Protein phosphatase 1 regulatory subunit 15 A (PPP1R15A) promoted the progression of gastric cancer by activating cell autophagy under energy stress
Source: J Exp Clin Cancer Res. 2025 Feb 13;44:52. doi: 10.1186/s13046-025-03320-y (PMC11823012; doi:10.1186/s13046-025-03320-y)
Supplement: Supplementary file 11 — Supplementary Material 11 [file 13046_2025_3320_MOESM11_ESM.docx]

**Supplementary Figure legends**

SFig. 1 The effects of glucose starvation on GC. (A) The mRNA expression of glucose metabolism-related genes under different glucose concentrations. (B-D) The proliferation (B), apoptosis (C), and cell cycle (D) of GC cells AGS and HGC-27 under energy stress.

SFig. 2 Energy stress-induced cell autophagy. (A) The expressions of autophagy-related protein expressions in AGS detected using Western blot. (B) Autophagosome and autolysosome formation detected in AGS using mRFP-GFP-LC3B adenovirus infection by laser scanning confocal microscopy. The red spots indicated autolysosomes, whereas the yellow spots represented autophagosomes.

SFig. 3 The HALLMARK gene set enrichment analyses of RNA-seq (A) and GSE13548 (B)

SFig. 4 C-Jun positively regulated PPP1R15A expression. (A) Dual-luciferase reporter assay after the transfection of the JUN overexpression vector (JUN) and empty vector (Vector) used as control. (B, C) The mRNA (B) and protein (C) expression levels of PPP1R15A and JUN after the transfection of the JUN overexpression vector and empty vector used as control.

SFig. 5 The protumor role of PPP1R15A in GC. (A, B) The mRNA and protein expressions of PPP1R15A after overexpression vector transfection in BGC-823 (A) and siRNAs transfection in SGC-7901 (B). (C, D) Cell proliferation ability after PPP1R15A overexpression (C) and knockdown (D). (E, F) Colony formation ability after PPP1R15A overexpression (E) and knockdown (F). (G, H) Cell apoptosis after PPP1R15A overexpression (G) and knockdown (H). (I, J) Cell cycle after PPP1R15A overexpression (I) and knockdown (J).

SFig. 6 PPP1R15A affected cell autophagy in GC. (A) Volcanic map of RNA-seq data of HGC-27 with stable PPP1R15A overexpression. The red dots indicated the upregulated genes, whereas the blue dots denoted the downregulated genes. (B) The GO enrichment analysis of DEGs. (C) The KEGG enrichment analysis of DEGs. (D) Volcanic map of Metabolite-seq data of HGC-27 with stable PPP1R15A overexpression. (E, F) Pathway enrichment analysis of differential primary (E) and secondary (F) metabolites. (G) Association between PPP1R15A and autophagy-related genes in TCGA-STAD. (H) Expressions of autophagy-related genes in different PPP1R15A expression groups in TCGA-STAD. (I, J) GO (I) and KEGG pathway (J) enrichment analyses on DEGs in different PPP1R15A expression groups in TCGA-STAD.

SFig. 7 PPP1R15A activated cell autophagy in GC. (A, B) Effects of PPP1R15A overexpression in BGC-823 (A) and knockdown in SGC-7901 (B) on autophagy-related protein expressions. (C, D) Effects of PPP1R15A overexpression in BGC-823 (C) and knockdown in SGC-7901 (D) on cell autophagy observed by laser scanning confocal microscope. Red spots indicated autolysosomes, whereas yellow spots denoted autophagosomes.

SFig. 8 PPP1R15A regulates autophagy under energy stress. (A) Effects of PPP1R15A knockdown on autophagy-related protein expressions in SGC-7901 after glucose deprivation for 24 h. (B) Effects of PPP1R15A knockdown on cell autophagy after glucose deprivation for 24 h in SGC-7901 observed by laser scanning confocal microscope.
